# Identification of key genes potentially associated with bladder cancer development by common plasticizers: an integrated transcriptomics and network toxicology study

**DOI:** 10.3389/fonc.2026.1784832

**Published:** 2026-07-01

**Authors:** Qiuqiu Zhang, Kailiang Xu, Zhongjun Chen, Zihui Li, Dong Zhao

**Affiliations:** 1 Department of Dermatology, Jingzhou Central Hospital, Jingzhou Hospital Affiliated to Yangtze University, Jingzhou, Hubei, China; 2 Hubei Provincial Clinical Research Center for Diagnosis and Therapeutics of Pathogenic Fungal Infection, Jingzhou, Hubei, China; 3 Department of Urology, Jingzhou Central Hospital, Jingzhou Hospital Affiliated to Yangtze University, Jingzhou, Hubei, China; 4 Department of Oncology, The Third People’s Hospital of Hubei Province, Affiliated Hospital of Jianghan University, Wuhan, Hubei, China; 5 Department of Rehabilitation, Tongji Hospital, Tongji Medical College, Huazhong University of Science and Technology, Wuhan, Hubei, China

**Keywords:** bladder cancer, machine learning, molecular docking, network toxicology, plasticizer

## Abstract

**Purpose:**

Bladder cancer (BCa) is a prevalent urological malignancy. The relationship between plasticizers and BCa remains to be elucidated. The present study aimed to investigate the potential relationship between BCa and plasticizers.

**Methods:**

BCa-related data were obtained from public databases. We performed simultaneous prediction of plasticizer toxicity, as well as identification of plasticizer−related genes (PRGs) and BCa−related target genes (BCRTGs). Key genes were then identified via machine learning and the expression of the screened genes was detected using qRT-PCR. We explored the effects of key genes in BCa tumorigenesis by gene set enrichment analysis (GSEA) and constructing a molecular regulatory network using Cytoscape software. Molecular docking and molecular dynamics (MD) simulation were employed to assess the binding affinity between plasticizers and key genes. Finally, the immune microenvironment of BCa was explored.

**Principal results:**

The comprehensive data set contains a total of 4,642 differentially expressed genes. In addition, 225 PRGs and 196 BCRTGs were obtained from various online databases, and then seven candidate genes were obtained. Functional analyses revealed key potential mechanisms in BCa, such as the cell differentiation-related pathways and PI3K-Akt signaling pathway. Furthermore, five key genes (CCNE1, KIT, BCL2, TGFBR2, FASN) were then identified. In comparison to normal cells, FASN and CCNE1 exhibited elevated expression levels, while KIT, BCL2, and TGFBR2 demonstrated reduced expression in BCa cells. GSEA revealed that a subset of genes were co-enriched in cell cycle regulation. Molecular regulatory network showed that key genes have shared microRNAs (miRNAs) and TFs between them. Molecular docking and MD simulations demonstrated favorable binding affinities between the proteins encoded by the five key genes and the 3 plasticizers. Further findings revealed dysregulated infiltration levels of immune cells, including activated B cells, in BCa.

**Major conclusions:**

Five genes (CCNE1, KIT, BCL2, TGFBR2, FASN) were identified. This study used bioinformatics and network pharmacology to generate hypotheses about the possible molecular mechanisms underlying the association between plasticizer exposure and BCa, offering insights for new therapeutic approaches.

## 1 Introduction

Bladder cancer (BCa) is one of the most common malignant tumors of the urinary system, ranking ninth in global cancer incidence. Approximately 614,298 new cases and 220,596 deaths were reported worldwide in 2022, making it the thirteenth leading cause of cancer-related mortality globally (1, 2). The risk factors for BCa include smoking, exposure to industrial chemicals, chronic bladder inflammation, and a family history of the disease. The pathogenesis of BCa involves complex molecular and genetic alterations (3, 4). Recent research has emphasized the importance of epithelial-mesenchymal transition (EMT) in the progression of BCa, characterized by the reduction of epithelial markers like E-cadherin and the increase of mesenchymal markers such as vimentin, which facilitate tumor invasion and metastasis (5). Kinesin family member 22 (KIF22) has been implicated in promoting BCa progression by activating cell division cycle-associated protein 3 (CDCA3). High expression levels of KIF22 have been correlated with advanced clinical stages and recurrence, indicating its potential as a prognostic marker and therapeutic target (6). Even with improvements in diagnostic and treatment methods, the prognosis of advanced BCa remains poor, with high rates of recurrence and progression presenting major challenges for clinical management (7). Given the public health burden and the need to improve therapeutic strategies, understanding the etiology and underlying molecular mechanisms of bladder cancer is critically important. Given the public health burden and the need for improved treatment strategies, understanding the etiology and underlying molecular mechanisms of bladder carcinogenesis is of paramount importance. Recent genomic studies have demonstrated that non-muscle-invasive bladder cancer (NMIBC) and muscle-invasive bladder cancer (MIBC) harbor distinct genetic driver features (8). Activating mutations in genes such as FGFR3 and PIK3CA are prevalent in NMIBC, whereas MIBC is predominantly characterized by TP53 inactivation and aberrations in the RB1 pathway (9). Furthermore, MIBC can be further classified into luminal, basal and neuronal molecular subtypes, which exhibit substantial differences in genetic alterations and clinical prognosis (10).

Recent epidemiological and toxicological studies have raised concerns about the role of environmental chemicals—particularly plasticizers—in the pathogenesis of BCa. Plasticizers, such as diethyl phthalate (DEP), dimethyl phthalate (DMP), and dioctyl phthalate (DOP), are widely used in the production of plastics and consumer goods, leading to ubiquitous human exposure through ingestion, inhalation, and dermal contact (11). These three phthalates were selected mainly because they are among the most frequently detected compounds in environmental and biomonitoring studies. The metabolites of these substances are primarily excreted through urine, resulting in direct and prolonged exposure to the urothelium of the bladder (12, 13). Furthermore, existing toxicological studies have suggested that they may exert endocrine-disrupting and genotoxic effects, which are potentially relevant to urinary tract tissues (14, 15). It should be clarified that we do not aim to establish organ-specific causality; instead, we intend to provide a biologically plausible theoretical basis for investigating a potential association with bladder cancer, grounded in the natural pathway of urinary excretion. Accumulating evidence indicates that these compounds and their metabolites possess endocrine-disrupting properties. They can perturb cellular signaling pathways and facilitate tumorigenesis in multiple organs. Given that these substances are primarily excreted via urine, resulting in direct exposure to the bladder mucosa, along with their endocrine-disrupting effects, we hypothesize that they may pose a potential risk for bladder cancer development (16). Although the association between plasticizer exposure and increased risk of BCa has been documented, the molecular targets and precise biological mechanisms mediating this relationship remain largely elusive.

Network toxicology has emerged as a powerful approach to elucidate the complex interactions between chemicals, genes, and biological pathways at the systems level. By constructing chemical-target-pathway networks and integrating multi-omics data, network toxicology allows for the identification of key molecular players and regulatory circuits implicated in chemical-induced toxicity and disease (17). Furthermore, molecular docking techniques facilitate the understanding of direct interactions between small molecules and protein targets, providing insights into binding affinities and the potential for chemical-induced modulation of gene function (18). The combination of these computational strategies enables a precise dissection of the mechanisms underlying environmental carcinogenesis (19).

This study utilizes BCa-related data obtained from The Cancer Genome Atlas (TCGA) and the Gene Expression Omnibus (GEO) databases. Relevant targets associated with plasticizers and BCa are identified through various online databases. Additionally, key genes related to plasticizers in BCa are evaluated using bioinformatics approaches, including machine learning methods. The potential mechanistic roles of these genes in BCa are also explored. The aim of this research is to generate hypotheses regarding the molecular mechanisms of these key genes and their potential relevance to the disease in the context of plasticizer exposure, and to provide new references for the precise diagnosis and personalized treatment of BCa patients.

## 2 Materials and methods

### 2.1 Data acquisition

The BCa transcriptome dataset of TCGA-BLCA was obtained from the University of California, Santa Cruz (UCSC) Xena database (https://xena.ucsc.edu/, accessed on December 4, 2025) as a training set comprising 409 BCa tumor samples and 19 normal tissue samples (20). The GSE13507 (platform: GPL6102) was obtained from the GEO database (https://www.ncbi.nlm.nih.gov/geo/, accessed on December 4, 2025); the database included 165 primary BCa samples and 68 paracancerous tissue samples (21). Samples were selected if labeled as “Primary bladder cancer”, “Bladder mucosae surrounding cancer”, “Multipotent Progenitor Cells (MPPs)”, and “Normal bladder mucosa”.

### 2.2 Acquiring common chemical components in plasticizers and predicting plasticizer toxicity

A comprehensive review of the extant literature and relevant databases, such as PubMed (https://pubmed.ncbi.nlm.nih.gov/, accessed on December 4, 2025), Google Scholar (https://scholar.google.com, accessed on December 4, 2025), and PubChem (https://pubchem.ncbi.nlm.nih.gov/, accessed on December 4, 2025) (22), was conducted to ascertain chemical components common to plasticizers. A comprehensive search, employing keywords such as “diethyl phthalate”, “dioctyl phthalate”, and “dimethyl phthalate”, was performed to identify relevant sources. In order to predict the toxicity of the three plasticizers, their full names were entered into the ProTox database (https://tox.charite.de, accessed on December 4, 2025), the SMILES structures of the three plasticizers were then entered in Canonical Smiles format, and TOX-Prediction was selected. Subsequently, the toxicity rank, toxicity category, prediction accuracy, toxicity target, and the three most similar compounds were obtained.

### 2.3 Acquisition of chemical component targets and disease-related targets

In order to obtain the plasticizer-related genes (PRGs), the STITCH (http://stitch.embl.de/, accessed on December 4, 2025) database was searched for “Diethyl phthalate”, “Dimethyl phthalate”, and “Dioctyl phthalate”, with the species restricted to “Homo sapiens”. Only targets with a confidence score ≥ 0.4 were retained. Separately, the SMILES structures of the three plasticizers were submitted to the SwissTargetPrediction database (http://swisstargetprediction.ch/, accessed on December 4, 2025), and “Predict Targets” was selected with species = “Homo sapiens”. Predicted target genes with a probability ≥ 0.5 were included. The target genes obtained by both methods were subsequently amalgamated using the ggvenn package (v 0.1.10) (23). The target genes of the three plasticizers were subsequently standardized with UniProt (https://www.uniprot.org, accessed on December 4, 2025) database as a reference. BCa-related target genes (BCRTGs) were obtained from GeneCards (https://www.genecards.org/, accessed on December 4, 2025) using the keyword “BCa”, and genes with a relevance score ≥ 50 were selected. Additionally, BCa-related genes were retrieved from the OMIM database (https://www.omim.org/, accessed on December 4, 2025) using the same keyword without applying an additional score threshold, as OMIM curates disease-associated genes based on literature evidence. The results from the two sources were subsequently combined and removed duplicates using the ggvenn package (v 0.1.10). The Cytoscape (v 3.10.3) software was subsequently employed to construct a network comprising three plasticizer chemistries-PRGs.

### 2.4 Differential gene expression analysis

Differentially expressed genes (DEGs) between the BCa and control groups in the TCGA-BLCA cohort were identified using the DESeq2 package (v 1.40.2) (24), The Benjamini−Hochberg (BH) method was used for multiple testing correction to control the false discovery rate (FDR),with the threshold of |log_2_fold change (FC)| > 1 and adj. p < 0.05. Furthermore, a volcano plot for DEGs was created using the ggplot2 package (v 3.4.1) (25) and labeled with the top ten gene names, while heatmap of top ten DEGs was generated using the ComplexHeatmap package (v 2.14.0) (26).

### 2.5 Functional analysis of candidate genes

The intersection of DEGs, PRGs and BCRTGs was determined using the ggvenn package, thereby identifying genes pertinent to plasticizers within BCa, which were subsequently designated as candidate genes. Thereafter, Gene Ontology (GO) and Kyoto Encyclopedia of Genes and Genomes (KEGG) enrichment analyses (adjusted p < 0.05) of the candidate genes were performed using the clusterProfiler package (v 4.15.0) (27) to elucidate the biological functions in which the candidate genes were implicated. Moreover, the Search Tool for the Retrieval of Interaction Genes/Proteins (STRING) database (https://www.string-db.org, accessed on December 4, 2025) was utilized to construct a protein-protein interaction (PPI) network (interaction score ≥ 0.4) to analyze protein-level interactions among candidate genes.

### 2.6 Discernment of key genes through machine learning algorithm and expression validation

In the TCGA-BLCA dataset, two distinct machine learning methodologies were utilized to ascertain genes that exhibited a high degree of association with BCa and plasticizers. Initially, least absolute shrinkage and selection operator (LASSO) regression analysis was conducted on candidate genes using glmnet package (v 4.1-8) (28). The optimal penalty parameter λ was selected as lambda.min based on 5-fold cross-validation, and genes with non-zero regression coefficients were retained. Simultaneously, support vector machine-recursive feature elimination (SVM-RFE) analysis was applied to gain feature genes with the highest accuracy rate utilizing the caret package (v 6.0-93) (29). A radial basis function kernel (svmRadial) was employed, and feature subsets were evaluated over a size range of 1 to 7; the optimal feature set was determined as the one yielding the highest cross-validation accuracy. The ggvenn package (v 0.1.10) was then used to identify the overlap between the aforementioned feature genes, resulting in candidate key genes selection. Furthermore, a comparative analysis of the expression profiles of these genes in TCGA-BLCA and GSE13507 was conducted between the BCa and control groups utilizing the Wilcoxon test. The candidate key genes that demonstrated significant differential expression between groups (p < 0.05) and a consistent trend across both TCGA-BLCA and GSE13507 were defined as key genes. To further perform expression validation of key genes, E-MTAB-1560 (affymetrix human gene 1.0 st array) was obtained from the ArrayExpress database (https://www.ebi.ac.uk/biostudies/arrayexpress, accessed on June 26, 2025). The dataset included 19 BCa tumor samples and 11 normal samples. The Wilcoxon rank-sum test was used to analyze the differences in the expression levels of key genes (p < 0.05). To investigate the differential expression profiles of hub genes across distinct invasive subtypes of Transitional Cell Carcinoma (TCC), we stratified TCC patients into NMIBC (Ta, Tis, T1) and MIBC (T2, T3, T4) subgroups based on pathological T stage. DESeq2-based differential expression analyses were performed for three comparison cohorts: NMIBC vs. normal, MIBC vs. normal, and MIBC vs. NMIBC to precisely characterize the dynamic expression alterations of these genes during early carcinogenesis and invasive progression (|log_2_FC| > 1 and adj. p < 0.05).

### 2.7 Construction of the exposure risk model for BCa patients

To develop an exploratory risk model for BCa patients, we employed expression data from hub genes alongside clinical information sourced from the TCGA-BLCA dataset. First, univariate Cox regression was applied to five genes and clinical covariates (age, sex, T stage, nodal status, grade). Variables with p < 0.1 were entered into multivariate Cox regression with backward stepwise selection. The final model included all five genes plus covariates that were significant (p < 0.05) or clinically relevant (age, sex, stage). The survminer package was then used to identify the optimal cutoff point for stratifying patients into high-risk and low-risk categories (30). Prognostic value was assessed using Kaplan-Meier curves with log-rank tests for overall survival (OS), and predictive accuracy was evaluated via time-dependent Receiver Operating Characteristic (ROC) curve analysis. Furthermore, we performed external validation in the GSE13507 dataset and conducted Kaplan–Meier analysis for each prognostic gene.

### 2.8 Cell culture and detection of core genes expression by qRT-PCR

To validate the expression patterns of the five key genes (CCNE1, KIT, BCL2, TGFBR2, FASN) identified from tissue datasets, we compared their expression levels in the bladder cancer cell line T24 and the normal urothelial cell line SV−HUC−1 using qRT−PCR. Human BCa cells (T24) and urinary tract epithelial cells (SV-HUC-1) were procured from the Institute of Cell Biology at the Chinese Academy of Sciences, located in Shanghai, China. These cell lines were cultured in Dulbecco’s Modified Eagle Medium (DMEM) supplemented with 10% fetal bovine serum (FBS) sourced from Invitrogen, USA, and 1% antibiotic solution. The cultivation process was conducted at 37 °C in a humidified incubator with an atmosphere of 5% CO_2_.

Total RNA was isolated from both cell groups utilizing the TRIzol reagent (Invitrogen, USA). Subsequently, the extracted mRNA was reverse transcribed into complementary DNA (cDNA) employing the SuperReal PreMix Plus (SYBR Green) (FP205-02, Tiangen, China). Quantitative real-time PCR (qRT-PCR) was then conducted to assess gene expression levels. The relative expression of the target genes was quantified using the 2^-ΔΔCT^ method. To ensure reliability, the experiment was conducted in triplicate. The expression levels of CCNE1, KIT, BCL2, TGFBR2, and FASN were normalized to GAPDH, which served as an internal control. The primer sequences utilized in this study are detailed in **Table 1**.

**Table 1:** Primer sequences of genes.

Gene	Primer Sequence(5`-3`)	
GAPDH	sense	CATCATCCCTGCCTCTACTGG
	antisense	GTGGGTGTCGCTGTTGAAGTC
CCNE1	sense	TTTCAGGGTATCAGTGGTGCG
	antisense	CCGCTGCTCTGCTTCTTACC
KIT	sense	TCACAACAACCTTGGAAGTAGTAG
	antisense	TTCATTCTCAGACTTGGGATAATC
BCL2	sense	ACATCGCCCTGTGGATGACT
	antisense	AGGGCCAAACTGAGCAGAGTC
TGFBR2	sense	TGAGAAGCCACAGGAAGTCTGT
	antisense	TGTCATTGCACTCATCAGAGCTAC
FASN	sense	GTCGGAGAACTTGCAGGAGTT
	antisense	GAGGCATCAAACCTAGACAGGTC

### 2.9 Analysis of subcellular and chromosomal localization

For a deeper understanding of the functional roles of key genes, sequence files were fetched in FASTA format from the gene database (https://www.ncbi.nlm.nih.gov/gene/, accessed on June 26, 2025), subcellular localization was predicted using the RNALocate database (http://rnalocate.org/, accessed on June 26, 2025), and visualization was performed with the ggplot2 package. Furthermore, annotation and mapping of the distribution of key genes on chromosomes were conducted with the RCircos package (v 1.2.2) (31).

### 2.10 Enrichment analysis of key genes

The gene set enrichment analysis (GSEA) was conducted to investigate the biological roles of crucial genes in BCa progression. The gene set “c2.cp.all.v7.0.symbols.gmt” from MSigDB was downloaded as a reference. For each key gene, all genes in the TCGA-BLCA dataset were ranked in descending order based on their Spearman correlation coefficient with the key genes via psych package (v 2.3.6) (32). Subsequently, GSEA was conducted employing clusterProfiler package (v 4.15.0), with Benjamini-Hochberg false discovery rate (FDR) < 0.25, |normalized enrichment score (NES)| > 1, and p < 0.05 considered significant.

### 2.11 Construction of plasticizer-key genes-pathway network

In order to investigate the relationship between “plasticizer-key genes-pathway”, plasticizer, key genes and key genes top5 GSEA enrichment pathway were imported into Cytoscape (v 3.10.3) software to construct plasticizer-key genes-key genes enrichment pathway network.

### 2.12 Molecular regulatory network construction

To elucidate regulatory mechanisms underlying key genes, miRDB database (http://mirdb.org/, accessed on December 6, 2025) was employed to identify potential microRNAs (miRNAs) targeting these genes. The top 10 miRNAs for each key gene were obtained, with the order of the list determined by the score value. Meanwhile, the KnockTF (http://www.licpathway.net/KnockTF/index.html, accessed on December 6, 2025) database was applied to predict transcription factors (TFs) of key genes, and the TFs were then identified according to the threshold of Fold Change > 2, and the top20 TFs were ranked according to the absolute value of log_2_FC to construct TFs-key gene relationship pairs. Subsequently, the key gene-miRNA network and the key gene-TFs network were constructed using Cytoscape (v 3.10.3).

### 2.13 Molecular docking and molecular dynamics simulation

In order to simulate the binding patterns between plasticizers and key genes, the three plasticizer structures were retrieved as SDF format files in PubChem (https://pubchem.ncbi.nlm.nih.gov/, accessed on December 7, 2025). The 2D structure files were converted to 3D structure in mol2 format files by ChemBio3D software (v 14.0.0) (33). The spatial structure information of the proteins encoded by five key genes (CCNE1, KIT, BCL2, TGFBR2, and FASN) was obtained from the Protein Data Bank (PDB) database (http://www.rcsb.org/, accessed on December 7, 2025) and processed using the AutoDockTools software (https://autodock.scripps.edu/, accessed on December 7, 2025) to prepare protein molecules. This process entailed the removal of water molecules and the subsequent addition of polar hydrogens. The resulting files were saved as.pdbqt files. Subsequent to this, molecular docking was performed using the AutoDock Vina software (34), and the binding energy was calculated along with the molecular docking results. PyMOL package (v 3.1.1) (35) was then used to visualize the docking results. The magnitude of the observed binding energy has been shown to indicate the strength of the interaction between the ligand and the receptor. A binding energy was ≤ -5.0 kcal/mol suggested potentially favorable binding.

To further verify the reliability of the molecular docking results, 100 ns molecular dynamics (MD) simulations were performed on the protein–ligand complexes using GROMACS (v 2024.4) (36) with the standard cascade protocol. The protein and small molecule were parameterized using the AMBER14SB and AMBER gaff force fields, respectively, to generate parameter and topology files. After setting periodic boundary conditions and determining the simulation box size, the system was solvated with water molecules. To maintain electroneutrality, some solvent water molecules were replaced with 0.15 mol/L Na^+^ and Cl^-^ ions. The entire system was energy-minimized using the steepest descent method. Pre-equilibration was carried out in two stages: first, temperature was stabilized under the NVT ensemble at 300 K for 100 ps; second, pressure was stabilized under the NPT ensemble at 1 bar for 100 ps. The root-mean-square deviation (RMSD) of the protein-ligand complex, the root-mean-square fluctuation (RMSF) of the protein, total energy, and the number of hydrogen bonds were computed.

### 2.14 Immune microenvironment analysis

To characterize the role of specific immune cell subsets in the progression of BCa, the infiltration abundance of 28 immune cell types in TCGA-BLCA (37) was analyzed using the ssGSEA algorithm in the GSVA package (v 1.50.0) (38), and a heatmap was plotted to display the results. Subsequently, the Wilcoxon test was utilized to compare the differences in immune cell infiltration abundance between the BCa and control groups (p < 0.05). Immune cells with significantly different infiltration levels were identified and termed differentially infiltrated immune cells. To examine the correlations among differentially infiltrated immune cells, as well as between key genes and these immune cells, Spearman correlation analyses were performed (p < 0.05, |correlation coefficient (cor)| > 0.3).

### 2.15 Statistical analysis

The R (v 4.2.3) software was employed to conduct the statistical analysis. For differential expression analyses, the Wilcoxon rank-sum test was used with a significance threshold of p < 0.05. It is noteworthy that **** represented p < 0.0001, *** represented p < 0.001, ** represented p < 0.01, * represented p < 0.05, and ns (no significant) represented p > 0.05.

## 3 Results

### 3.1 Chemical information of three plasticizers and toxicological study

**Figure 1:**
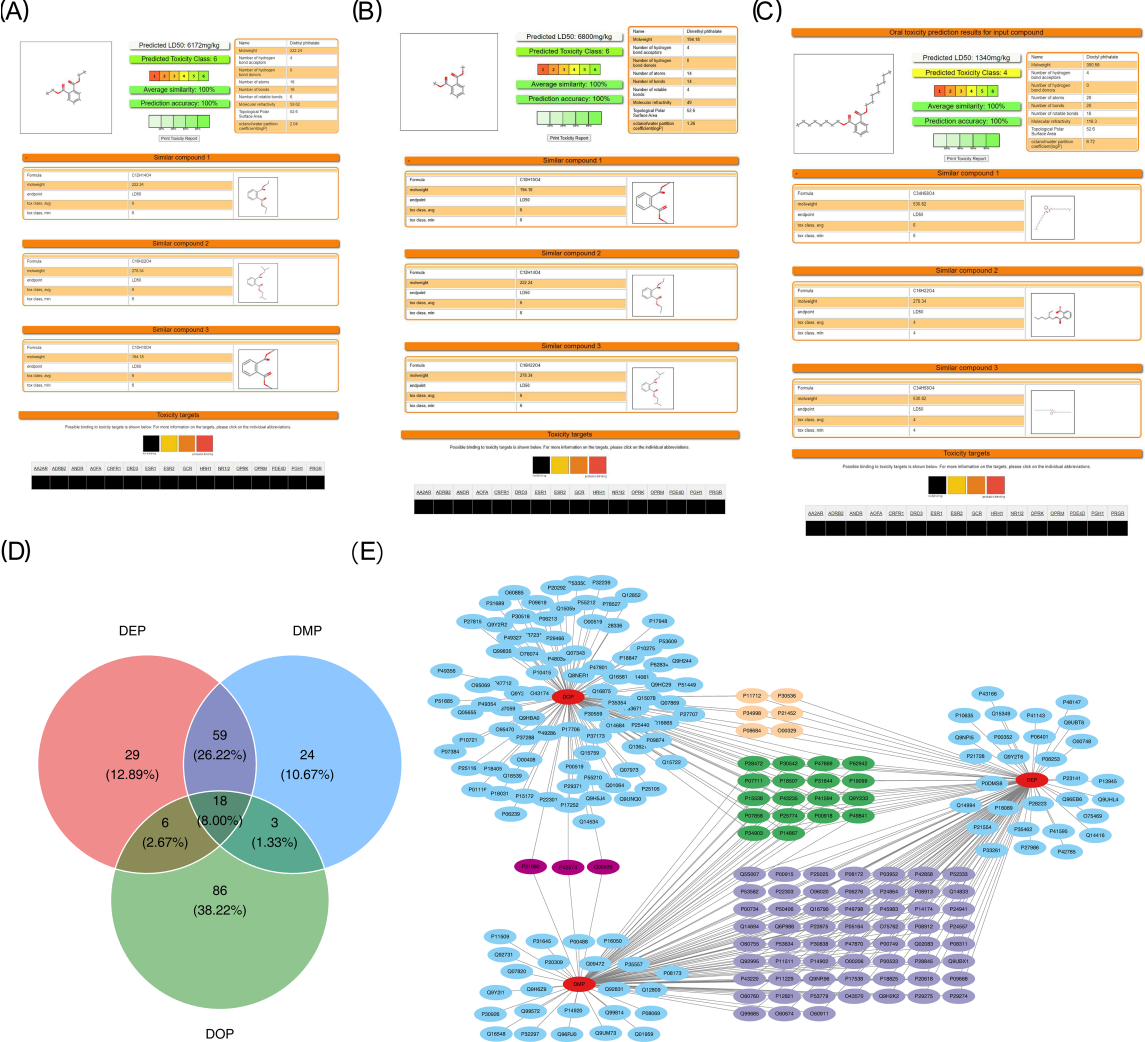
Chemical information of plasticizers, toxicological study, and determination of targeted specific genes. **(A–C)** Toxicity prediction results of Diethyl phthalate, Dimethyl phthalate and Dioctyl phthalate in ProTox database. The toxicity prediction results from the ProTox database included: the structure of the predicted compound, basic information such as molecular weight, the three compounds with the most similar structures, and the toxicity targets, which were protein targets associated with adverse drug reactions and toxic effects. **(D)** Venn diagram of target genes for three plasticizers. Eighteen target genes were identified. **(E)** Chemical composition of three plasticizers - PRGs network. The red nodes in the figure represented plasticizers, the green nodes represented target genes shared by three plasticizers, the purple nodes represented target genes shared by plasticizers DMP and DEP, the six flesh-colored nodes represented target genes shared by plasticizers DOP and DEP, the three magenta nodes represented target genes shared by plasticizers DMP and DOP, and the blue nodes represented target genes unique to the three plasticizers.

The chemical specifications of three plasticizers were listed in **Table 2**. This encompasses their SMILES structures, molecular weights and chemical formulas. A thorough investigation was conducted by systematically exploring a range of databases to acquire relevant data concerning the toxicity of the three compounds that exhibited the greatest similarity to DEP, DMP, and DOP (**Figures 1A–C**). ProTox toxicity prediction results revealed that diethyl phthalate (DEP) is categorized as toxicity Class 4 (harmful if swallowed, prediction accuracy: 0.70), with predicted molecular targets including estrogen receptors and androgen receptors. Dimethyl phthalate (DMP) is also classified under Class 4 (prediction accuracy: 0.70) and exhibits analogous nuclear receptor interaction profiles. Dioctyl phthalate (DOP) is assigned to Class 3 (toxic if swallowed, prediction accuracy: 0.54), accompanied by additional predictive hepatotoxicity. Although these toxicity classifications do not directly indicate carcinogenic potential, the predicted disruption of hormonal signaling pathways has been mechanistically linked to tumorigenesis in hormone-sensitive tissues, including the urinary bladder.

**Table 2:** Chemical composition of three plasticizers.

Plasticizer	Chemical formula	SMILES structure	MW (g/mol)
Diethyl phthalate (DEP)	C12H14O4	CCOC(=O)C1=CC=CC=C1C(=O)OCC	222.24
Dimethyl phthalate(DMP)	C10H10O4	COC(=O)C1=CC=CC=C1C(=O)OC	194.18
Dioctyl phthalate (DOP)	C24H38O4	CCCCCCCCOC(=O)C1=CC=CC=C1C(=O) OCCCCCCCC	390.6

### 3.2 Three chemical components specific genes targeted

The target genes of the three plasticizers were identified through the STITCH database and the SwissTargetPrediction database. Specifically, there were 112 target genes for DEP, 104 target genes for DMP, and 113 target genes for DOP. A total of 225 PRGs were identified through the consolidation of target genes from the three plasticizers and the removal of duplicate genes (**Supplementary Table 1**). Interestingly, a comparative analysis revealed a total of 18 target genes shared by the three plasticizer components (**Figure 1D**). The identified target proteins represented targets whose exposure to plasticizers might lead to BCa progression. Further exploration of these target molecules might help contribute to the development of strategies to mitigate negative health effects linked to exposure to plasticizers (**Figure 1E**).

### 3.3 Identification of BCRTGs

A total of 84 BCa-related target genes were retrieved from OMIM database, and 138 related target genes (Relevance.score ≥ 50) were screened from the Genecards database. The target genes from these two sources were then combined and de-duplicated to ultimately obtain 196 BCRTGs (**Supplementary Table 2**).

### 3.4 Identification of DEGs and candidate genes and their functional annotation

**Figure 2:**
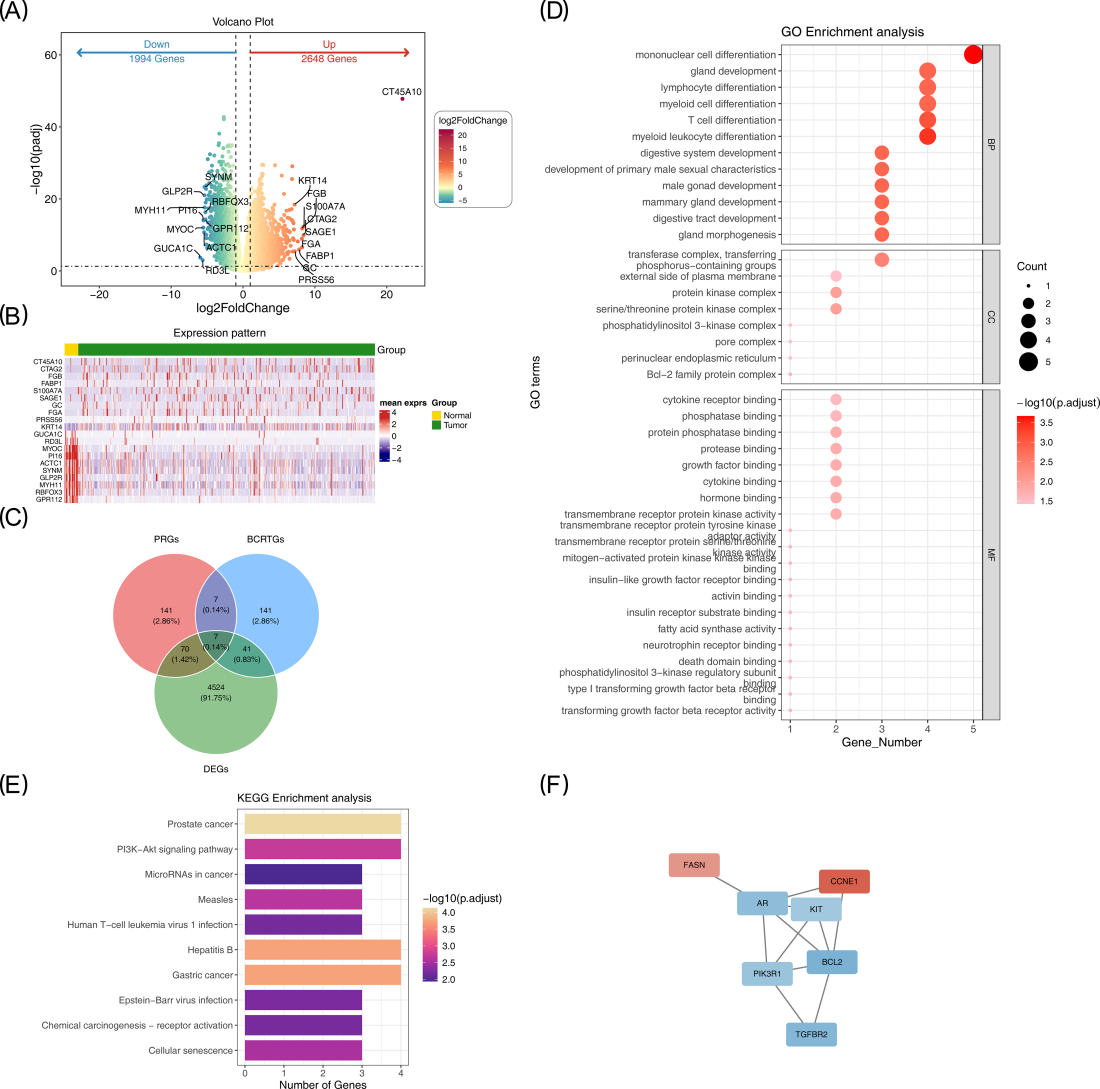
Identification of DEGs and candidate genes and their functional annotation. **(A)** Volcano plot of differentially expressed gene distribution between BCa and Normal samples. Red: up-regulated gene; Blue: Down-regulated gene. The top 10 up-regulated genes and the top 10 down-regulated genes were labeled. **(B)** Heatmap of differentially expressed gene distribution between BCa and Normal samples. Green: BCa groups; Yellow: Normal groups. The color of the heatmap indicated the expression level of the gene in the sample. **(C)** Venn diagram for identifying candidate genes. 7 candidate genes were identified. **(D)** Bubble plot of GO enrichment analysis for candidate genes. The size of the bubble represented the number of enriched genes; the color of the bubble represented the significance of enrichment. **(E)** Bar chart of KEGG enrichment analysis of candidate genes. The color of the bar represented the enrichment of p-values. **(F)** PPI network construction diagram of candidate genes. The genes marked with boxes were network nodes, and the gray lines between nodes were the edges of the network.

To identify genes associated with plasticizer-affected BCa, 4,642 DEGs in BCa, including 1,994 down-regulated and 2,648 up-regulated genes, were first obtained from TCGA-BLCA. These DEGs were mapped in a volcano plot (**Figure 2A**).The volcano plot contained labels indicating the top 10 DEGs that were up- or down-regulated according to log_2_FC ordering. Subsequently, their expression heatmaps were plotted as a proxy for DEGs (**Figure 2B**). Taking the intersection of DEGs, PRGs and BCRTGs resulted in seven candidate genes: “CCNE1”, “PIK3R1”, “KIT”, “BCL2”, “TGFBR2”, “FASN”, and “AR” (**Figure 2C**).

Furthermore, a significant enrichment of candidate genes was observed in 550 GO entries, encompassing 484 biological processes (BPs), 8 cellular components (CCs), and 258 molecular functions (MFs) (adj. p < 0.05) (**Supplementary Table 3**). The top BPs included “mononuclear cell differentiation”, “T cell differentiation”, “lymphocyte differentiation” and “myeloid cell differentiation”. The first five of these CCs include “transferase complex, transferring phosphorus-containing groups”, “serine/threonine protein kinase complex”, “protein kinase complex”, “Bcl-2 family protein complex” and “perinuclear endoplasmic reticulum”. And the first five of these MFs include “transmembrane receptor protein kinase activity”, “hormone binding”, “cytokine binding”, “growth factor binding” and “protease binding” (**Figure 2D**). Furthermore, KEGG enrichment analysis revealed the enrichment of 50 pathways (adj. p < 0.05) (**Supplementary Table 4**), and top ten pathways include “PI3K-Akt signaling pathway”, “Prostate cancer”, and “Cellular senescence” (**Figure 2E**). In the PPI network, a complex network comprising seven nodes and 11 edges, each node represents a specific gene, and each connecting line symbolized protein interactions between particular genes (**Figure 2F**). Among them, AR and BCL2 showed frequent interactions with proteins corresponding to other candidate genes.

### 3.5 Machine learning and expression validation identify key genes

**Figure 3:**
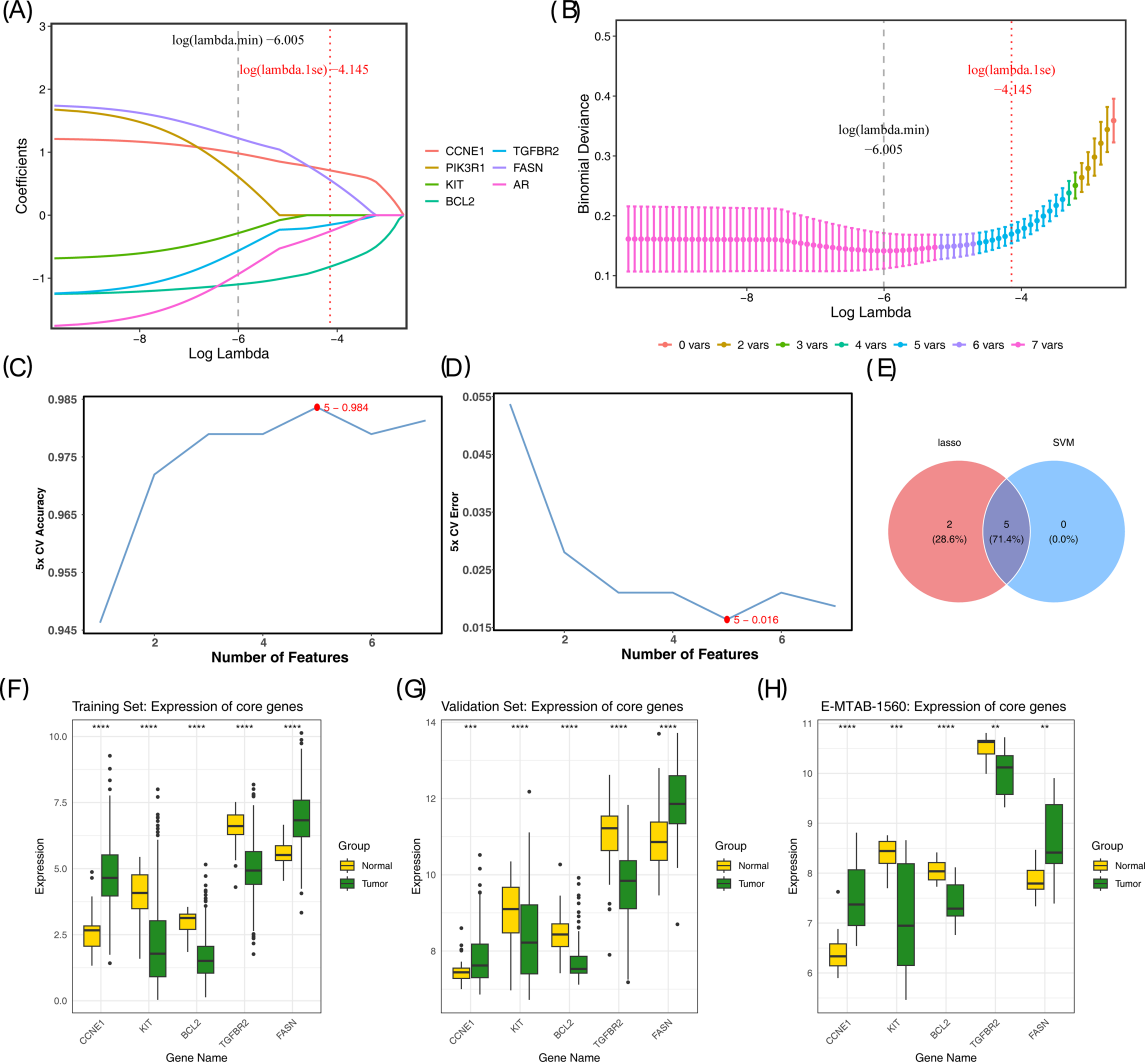
Identification of key genes. **(A)** LASSO regression coefficient path diagram. The gray dashed line marked the lambda. min with the smallest cross validation error as 0.00246, and the red dashed line marked the lambda. 1se with an error within 1 standard error as 0.01585. **(B)** LASSO cross validation error curve. The gray dashed line marked the lambda. min with the smallest cross validation error as 0.00246, and the red dashed line marked the lambda. 1se with an error within 1 standard error as 0.01585. **(C)** Accuracy Curve of Cross Validation. **(D)** Error rate curve of cross validation. **(E)** Venn diagram of key genes. 5 key genes were identified. **(F–G)** Validation of key gene expression levels in the training set and Validation set. Green, Tumor group; Yellow, Normal group. ***P<0.001; ****P<0.0001. **(H)** Verification of E-MTAB. **P<0.01; ***P<0.001; ****P<0.0001.

According to the analysis of LASSO regression algorithm, when the optimal λ value was 0.00246, the error rate of the model was minimized and seven candidate feature genes were obtained (**Figures 3A, B**). SVM-RFE screened five candidate characteristic genes (**Figures 3C, D**). Genes identified by two algorithms were subsequently intersected to identify five candidate key genes: “CCNE1, KIT, BCL2, TGFBR2 and FASN” (**Figure 3E**). The expression of these genes was then examined in two datasets. Results showed that all five candidate key genes were consistently expressed in both datasets and had significant intergroup differences (p<0.05). Among the genes under consideration, “CCNE1” and “FASN” demonstrated significantly elevated expression levels in tumor group. In contrast, remaining three genes exhibited notably higher levels of expression in the control group (**Figures 3F, G**). Moreover, consistent results were obtained in E-MTAB-1560, further demonstrating the reliability of the findings at the expression level (**Figure 3H**). These results confirm that the differential expression of the five key genes was reproducible across multiple independent datasets. Ultimately, these 5 genes were considered key genes for inclusion in subsequent analyses. In addition, stratified analyses demonstrated that the five hub genes shared identical expression trends in both NMIBC and MIBC compared with normal bladder tissues: CCNE1 and FASN were significantly upregulated, while KIT, BCL2 and TGFBR2 were markedly downregulated. Such consistent findings further validated the robustness of these five genes as pan-subtype diagnostic biomarkers for bladder cancer. Meanwhile, apart from CCNE1, none of the five genes enabled significant molecular discrimination between NMIBC and MIBC subtypes, indicating that these biomarkers mainly functioned to distinguish malignant lesions from normal tissues rather than predict muscular invasive status (**Supplementary Table 5**). This observation was consistent with the biological nature of TCC originating from a uniform pathological source and suggested that plasticizers might initiate TCC development via shared mechanisms including dysregulated cell cycle control (CCNE1/FASN) and disrupted apoptosis-related signal transduction (KIT/BCL2/TGFBR2), independent of tumor invasive depth.

### 3.6 Prognostic model of BCa

**Figure 4:**
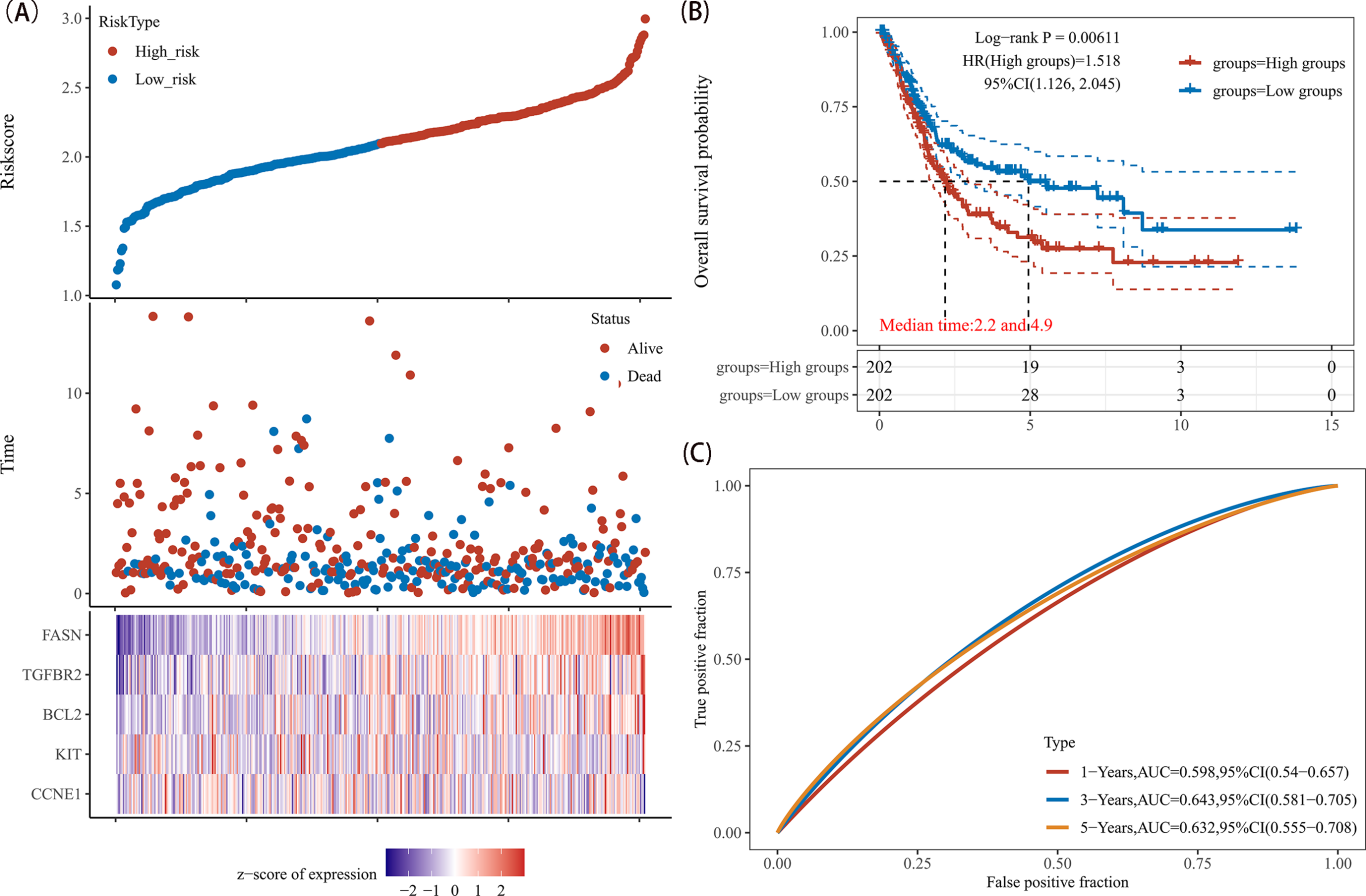
Prognostic model of BCa. **(A)**Distribution of patient risk scores (top), patient survival status and follow−up time (middle), and z−score–normalized expression heatmap of the five signature genes (bottom). **(B)** Kaplan–Meier overall survival curves for high−risk (red) versus low−risk (blue) groups. **(C)** Time−dependent receiver operating characteristic (ROC) curves for the risk model at 1, 3 and 5 years.

The risk score was calculated as the linear combination of the normalized expression value of each hub gene multiplied by its corresponding regression coefficient (β) derived from the multivariate Cox regression model. The regression coefficient quantifies the independent prognostic contribution of each gene: a positive coefficient indicates that higher gene expression is associated with increased mortality risk, while a negative coefficient indicates a protective effect. The risk score formula is defined as follows: Risk score = (-0.0881) * CCNE1 + (-0.0586) * KIT + (0.1051) * BCL2 + (0.1235) * TGFBR2 + (0.2702) * FASN. Based on this risk score calculation, the samples were categorized into high-risk and low-risk groups, with the distribution depicted in **Figure 4A**. The Kaplan-Meier survival curves indicated that the high-risk group had a significantly lower overall survival rate than the low-risk group (p < 0.01) (**Figure 4B**), and this conclusion also applied to each prognostic gene (**Supplementary Figure 1**). The time-dependent Receiver Operating Characteristic (ROC) curve showed Area Under the Curve (AUC) values of 0.73 at 1 year, 0.71 at 3 years, and 0.69 at 5 years in the TCGA-BLCA cohort (**Figure 4C**).

### 3.7 Results of qRT-PCR

**Figure 5:**
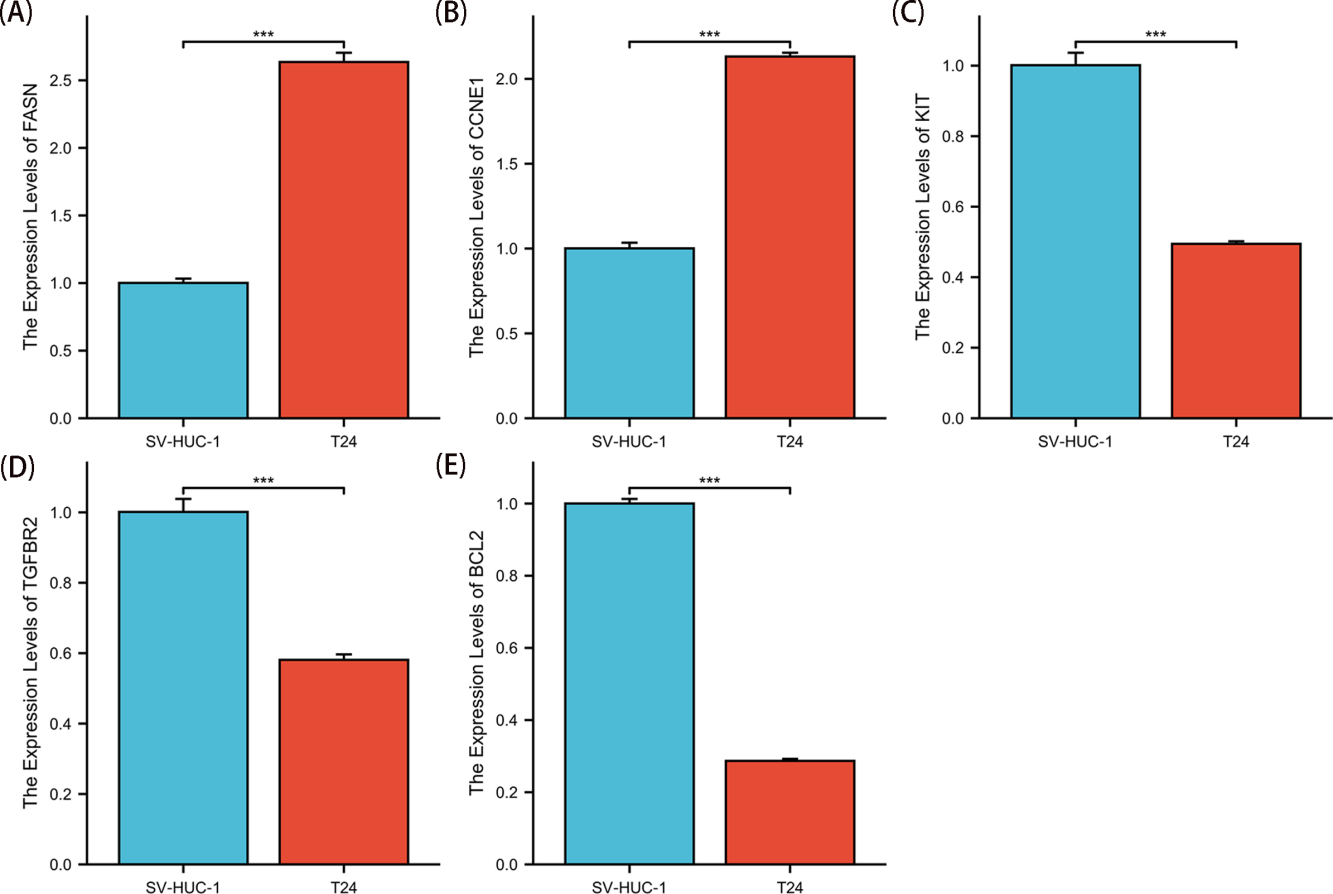
Comparative expression of selected genes in SV−HUC−1 and T24 cell lines. Bar graphs show relative expression levels of FASN **(A)**, CCNE1 **(B)**, KIT **(C)**, TGFBR2 **(D)**, and BCL2 **(E)** in non−tumorigenic SV−HUC−1 (blue) versus bladder cancer T24 (red) cells. **P<0.01; ***P<0.001; ****P<0.0001.

The expression of CCNE1, KIT, BCL2, TGFBR2 and FASN in the T24 bladder cancer cell model and the SV-HUC-1 immortalized normal urothelial cell model was detected using qRT-PCR. In comparison to SV-HUC-1 cells, FASN and CCNE1 exhibited elevated expression levels, while KIT, BCL2, and TGFBR2 demonstrated reduced expression in these specific cell models (**Figures 5A–E****).**

### 3.8 Localization and enrichment analysis of key genes

**Figure 6:**
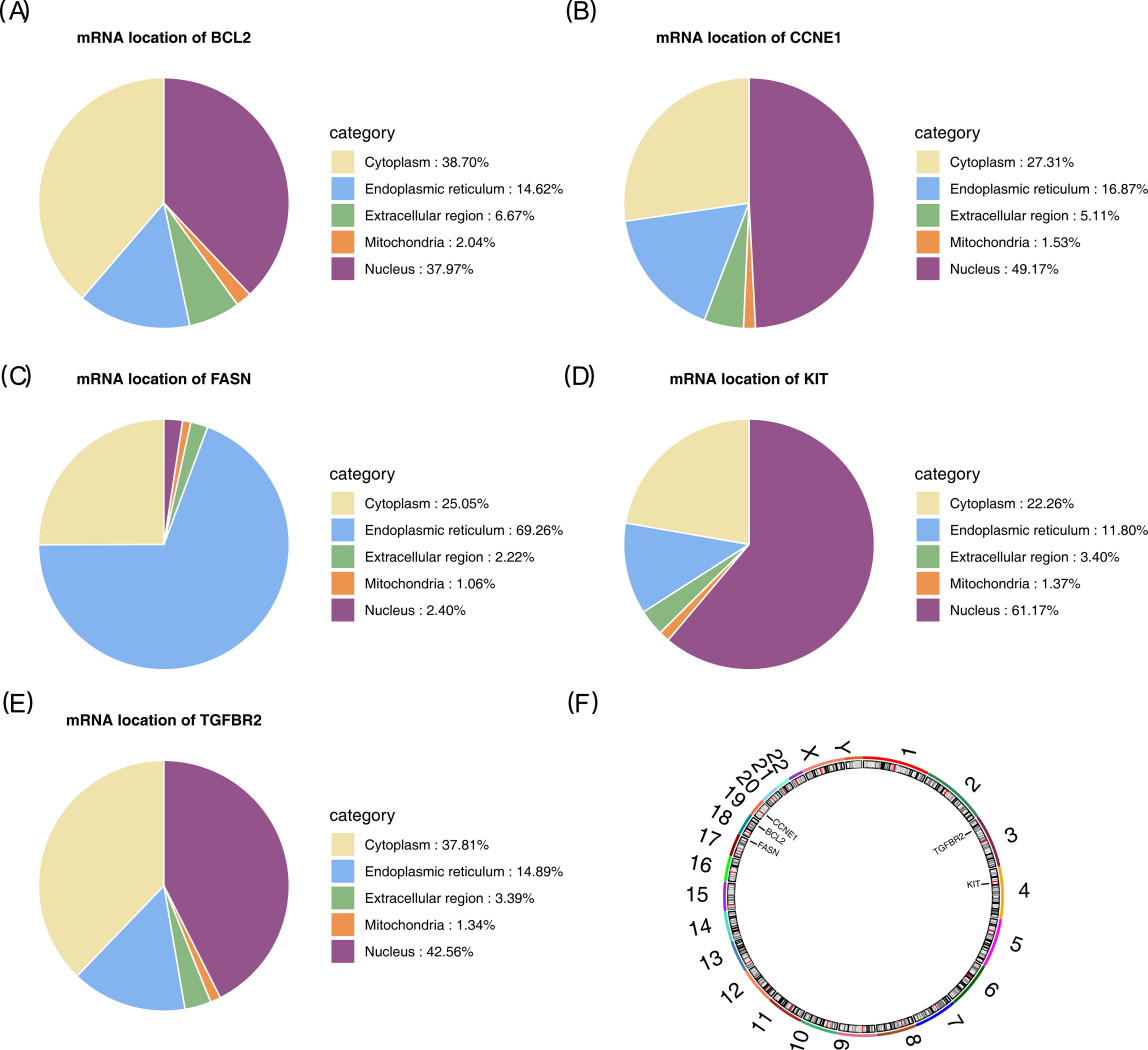
Localization of key genes. **(A–E)** Subcellular localization of key genes. The different color module sizes in the pie chart corresponded to different subcellular localization prediction probability values, and the labels on the right side of the pie chart indicated the subcellular localization positions and prediction probability values. **(F)** Chromosome localization of key genes.

Predictions of subcellular localization showed that BCL2, CCNE1, KIT and TGFBR2 were predominantly localized in the nucleus, and FASN was predominantly localized in the endoplasmic reticulum (**Figures 6A–E**). Chromosomal localization analysis demonstrated that FASN, BCL2, and CCNE1 were clustered on chromosomes 17, 18, and 19, respectively, while TGFBR2 and KIT were localized to chromosomes 3 and 4, respectively (**Figure 6F**).

**Figure 7:**
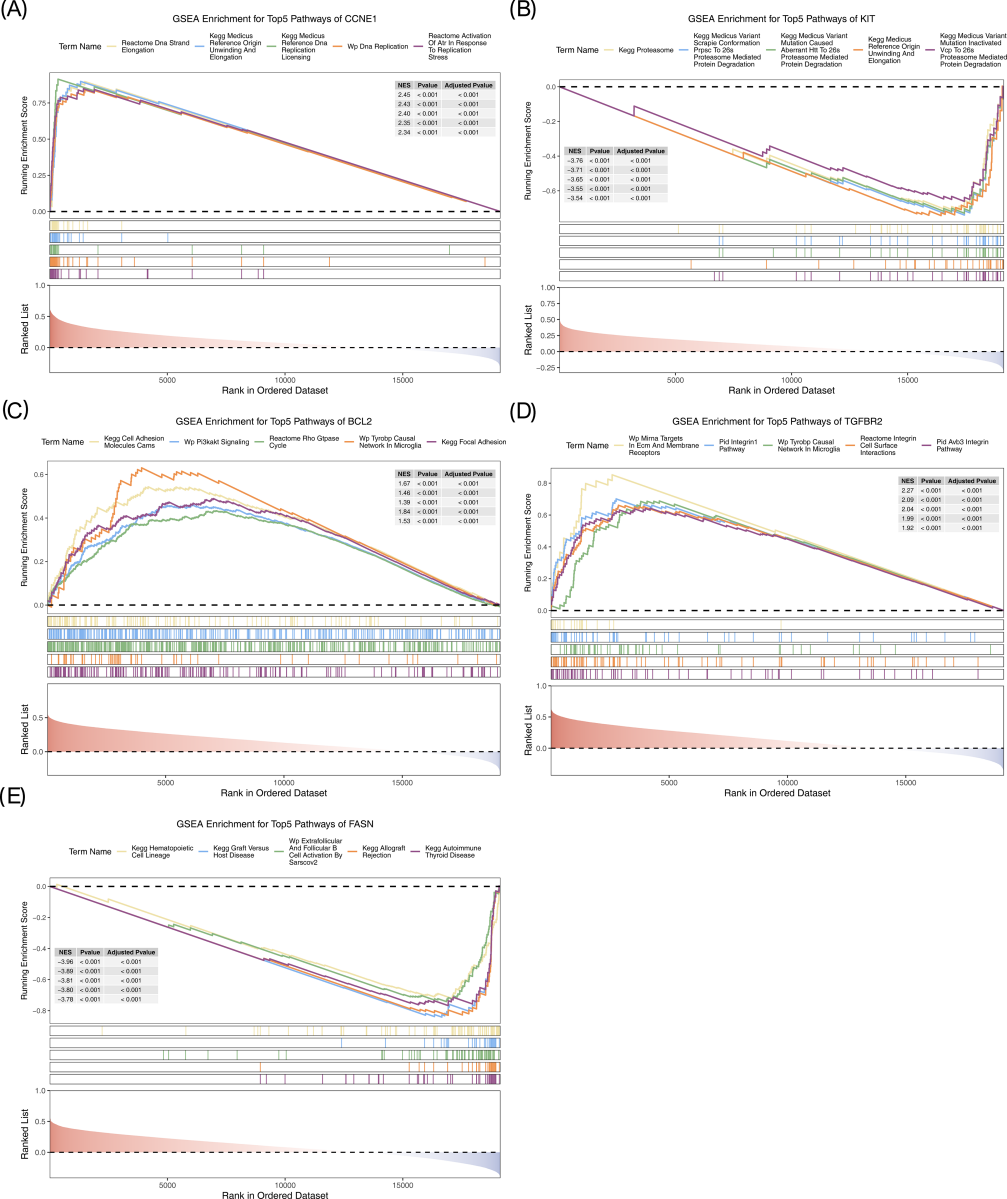
GSEA plots of top 5 enriched pathways for indicated genes. **(A)** Top five positively enriched pathways in CCNE1-high signature. **(B)** Top five negatively enriched pathways in KIT-high signature. **(C)** Top five positively enriched pathways enriched in BCL2-high signature. **(D)** Top five positively enriched pathways enriched in TGFBR2-high signature. **(E)** Top five negatively enriched pathways depleted in FASN-high signature. Each panel displays running enrichment score curves, ranked gene lists and corresponding normalized enrichment score (NES), P-value and adjusted P-value for each pathway.

The GSEA results showed that CCNE1, KIT, BCL2, TGFBR2 and FASN were enriched in 594, 286, 542, 986 and 889 pathways, respectively (**Figures 7A–E**; **Supplementary Table 6**). In particular, the enriched pathways of these genes focus on cell cycle regulation, DNA replication and genome stability, signaling and cell function, immune and inflammatory responses, and tumorigenesis and development. For example, CCNE1 and KIT were co-enriched in DNA strand replication and elongation-related pathways. And tumor-associated PI3K-Akt signaling pathway was the most relevant pathway for BCL2. Integrin α1 and integrin αVβ3 pathways were highly TGFBR2-relevant pathways. Besides, FASN played an important role in T-cell regulation and immunity systems. These commonalities reflect the diverse roles of these key genes in cellular physiology and pathology, particularly their importance in maintaining normal cellular functions and responding to intra- and extracellular stresses.

### 3.9 Construction of plasticizer-key genes-pathway network and molecular regulatory network

**Figure 8:**
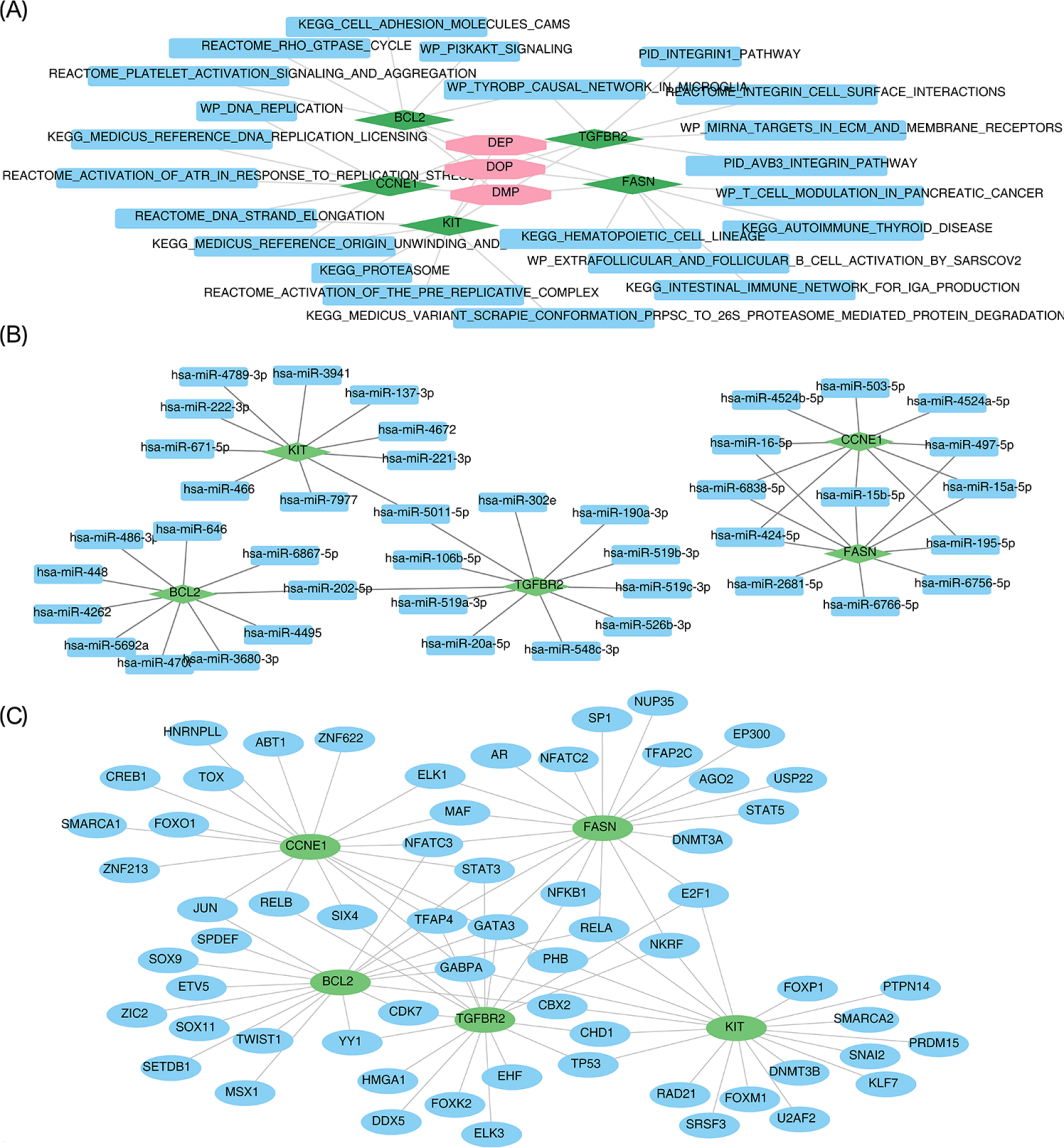
The pathway network and molecular regulatory network of key genes. **(A)** Plasticizer-key gene-key gene enrichment pathway network. The nodes marked in red represented plasticizers, the nodes marked in green represented key genes, and the nodes marked in blue represented pathways enriched by key genes. **(B)** Key genes-miRNA network. The green diamond nodes represented key genes, while the blue square nodes represent miRNAs. The gray lines between nodes indicated the regulatory relationship between genes and miRNAs. **(C)** TF-Key genes network. The green diamond nodes represented key genes, while the blue square nodes represent TF. The gray lines between nodes indicated the regulatory relationship between genes and TF.

As indicated by the plasticizer-key gene-key gene enrichment pathway network, five key genes demonstrated a high degree of association with DEP, DOP, DMP. Furthermore, the enrichment pathway encompassed an array of biological processes, including cell cycle, cell signaling, DNA replication and repair, and apoptosis (**Figure 8A**). Molecular regulatory networks offered additional understanding of the factors regulating CCNE1, KIT, BCL2, TGFBR2, and FASN. We obtained the top 10 miRNAs for each key gene, sorted by Score value. A total of 42 key miRNAs were identified as modulators of key genes. Among them, KIT shared hsa-miR-5011-5p with TGFBR2, BCL2 shared hsa-miR-202-5p with TGFBR2, while CCNE1 and FASN shared multiple miRNAs, such as hsa-miR-16-5p (**Figure 8B**). Subsequently, a total of 62 key TFs were identified as modulators of key genes (such as STAT3 and RELA) (**Figure 8C**). Such complex molecular interactions provide a systems-level perspective on the potential of precision medicine in disease management and may represent novel therapeutic targets for modulating the complex signaling pathways that lead to BCa progression.

### 3.10 Molecular docking and MD simulations

**Figure 9:**
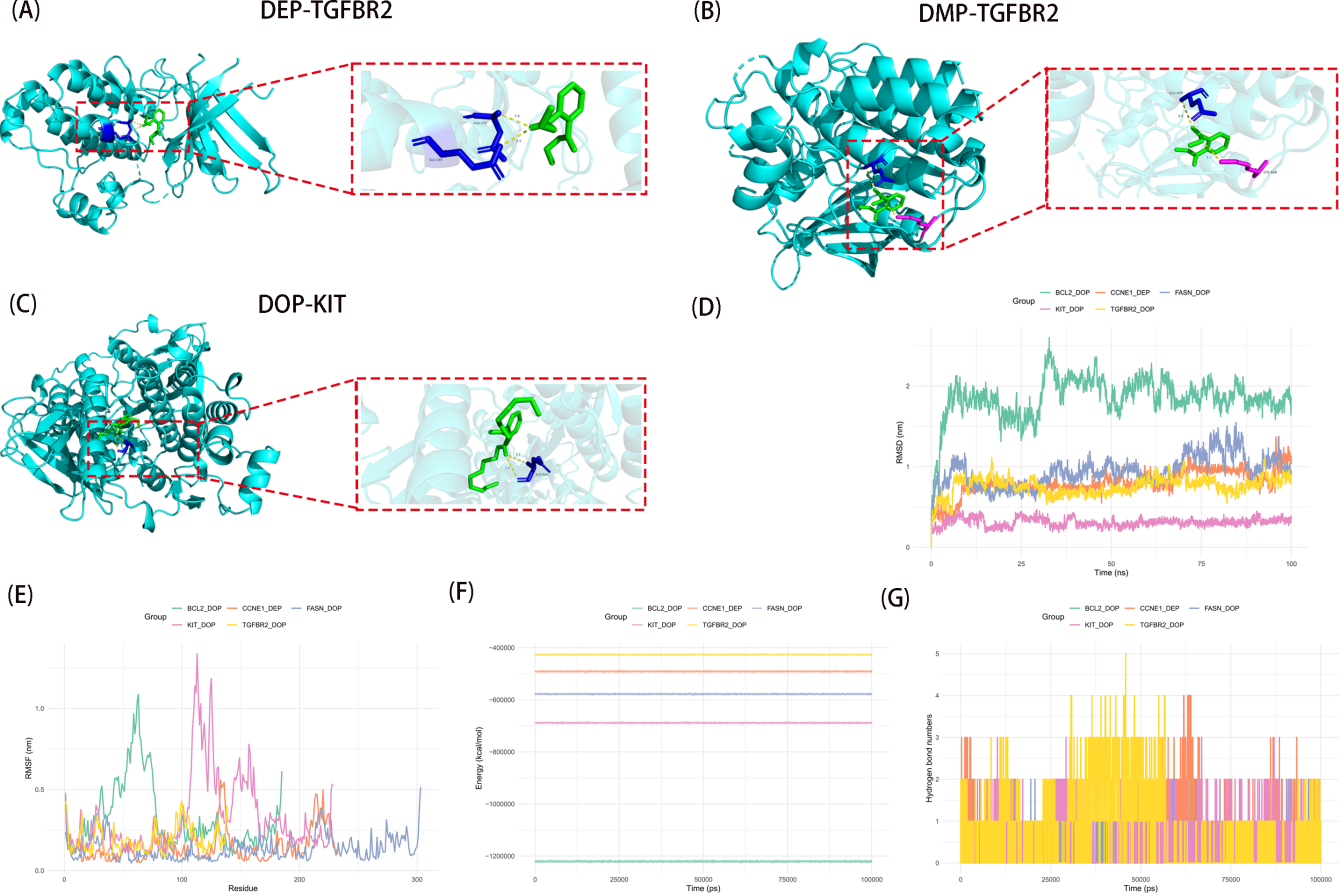
Molecular docking and molecular dynamics (MD) simulation analysis of ligand-protein complexes. Schematic diagrams of the TGFBR2-DEP **(A)**, TGFBR2-DMP **(B)**, and KIT-DOP **(C)** molecular docking models. Light blue represented the protein structure, green represented the small molecule structure of the plasticizer, yellow dashed lines represented hydrogen bonds, and dark blue represented the two residues on the protein that were connected by hydrogen bonds, with the residue names labeled next to them. **(D)** Root mean square deviation (RMSD) trajectories of protein-ligand complexes over a 100 ns MD simulation, illustrating structural stability. **(E)** Root mean square fluctuation (RMSF) values per residue, indicating flexibility changes upon ligand binding. **(F)** Potential energy profiles of the different protein-ligand systems during the simulation, reflecting system stability. **(G)** Hydrogen bond number variation over time for each complex, demonstrating dynamic interaction stability during MD simulations.

The proteins CCNE1 (PDB ID: 1W98), KIT (PDB ID: 1PKG), BCL2 (PDB ID: 1G5M), TGFBR2 (PDB ID: 1KTZ), and FASN (PDB ID: 2JFD) were included in our research. **Table 3** illustrated the minimum docking binding energies of DEP, DMP, and DOP in complex with the five key genes. The results indicated that all three plasticizers exhibited favorable binding affinities toward the five key genes. For example, the lowest binding energy of DEP to TGFBR2 was -6.6 kcal/mol (**Figure 9A**). The lowest binding energy of DMP to TGFBR2 was -5.9 kcal/mol (**Figure 9B**). Where the lowest docking binding energy of DOP and KIT was -7.2 kcal/mol (**Figure 9C**), indicating that these complexes formed stable dockings. Subsequently, MD simulations were performed for the binding of BCL2, FASN, KIT, and TGFBR2 with DOP, as well as for CCNE1 with DEP.

**Table 3:** Minimum docking binding energy of three plasticizers and key gene complexes.

Plasticizer	CCNE1	KIT	BCL2	TGFBR2	FASN
DEP	-5.3	-6.1	-5.7	-6.6	-5.5
DMP	-5.2	-5.5	-5.6	-5.9	-5.5
DOP	-5.1	-7.2	-5.8	-6.8	-6.3

The RMSD analysis revealed that the BCL2-DOP complex exhibited an initial sharp increase in RMSD, which then stabilized, while the other complexes maintained relatively stable fluctuations throughout the simulation (**Figure 9D**). Higher RMSF values were observed for BCL2-DOP and KIT-DOP, indicating enhanced flexibility in these binding regions (**Figure 9E**). The total energy of all systems remained stable with minimal fluctuation, suggesting no significant energy accumulation or dissipation during the simulation (**Figure 9F**). The number of hydrogen bonds between the compounds and proteins is shown in **Figure 9G**. Notably, the TGFBR2-DOP complex displayed considerable variation in hydrogen bond count, reaching a maximum of 5. The results suggest favorable binding affinities between the five key genes and the three plasticizers (binding energy ≤ -5.0 kcal/mol for all complexes), and that plasticizers may induce BCa by targeting the biological pathways that these genes were involved. Notably, the molecular dynamics simulation only preliminarily assessed dynamic stability and was insufficient for full conformational sampling or long-term stability evaluation. Longer simulations or experimental validation are needed for confirmation.

### 3.11 Analysis of the immune microenvironment in BCa

**Figure 10:**
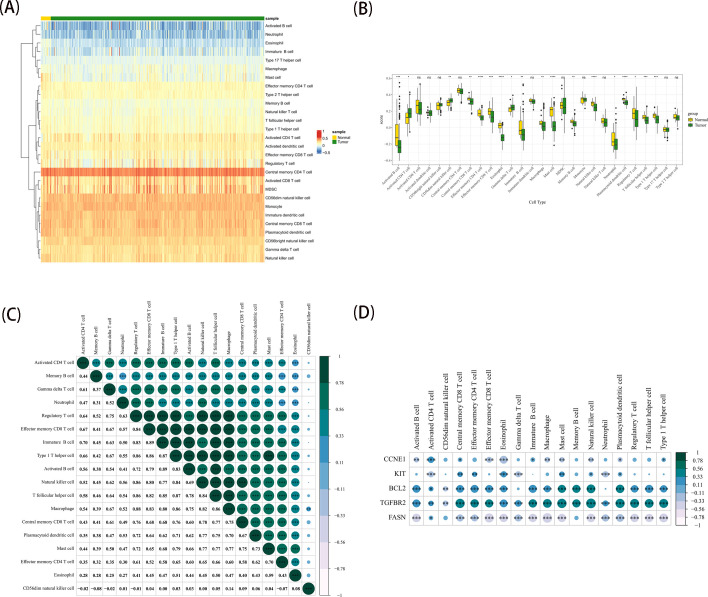
Immune-cell infiltration landscape and associations with hub genes. **(A)** Hierarchical clustered heatmap of inferred immune-cell enrichment scores across all samples. Columns represent individual samples annotated as tumor or normal; rows denote immune-cell subsets. Color scale indicates relative enrichment, highlighting group-specific infiltration patterns. **(B)** Boxplots comparing immune-cell enrichment scores between tumor and normal groups for each cell type. Central line denotes the median; boxes show the interquartile range (IQR); whiskers indicate 1.5×IQR. Differences illustrate tumor-associated remodeling of the immune microenvironment. **(C)** Pairwise correlation matrix among immune-cell subsets. Circle size and color encode the strength and direction of Spearman correlations; numeric labels display correlation coefficients, revealing coordinated or antagonistic infiltration behaviors. **(D)** Bubble plot of correlations between hub gene expression (CCNE1, KIT, BCL2, TGFBR2, FASN) and immune-cell enrichment scores. Bubble size reflects the absolute correlation magnitude, and color indicates positive or negative associations, linking gene activity to specific immune-cell contexts.

Immune cell infiltration is closely associated with BCa, influencing disease severity and immune responses. **Figure 10A** visualizes the immune infiltration profiles of 28 immune cell types in BCa and control groups from TCGA-BLCA. Marked disparities in infiltration levels of 17 immune cell types were noted between the BCa and control groups (p < 0.05). For example, compared with the control group, the infiltration scores of activated B cells (p < 0.001) and effector memory CD4 T cells (p < 0.001) were lower in the BCa group (**Figure 10B**). Further studies revealed a significant positive correlation between immature B cells and activated B cells (p < 0.001, cor = 0.89) (**Figure 10C**). Among biomarkers, TGFBR2 exhibited positive correlation with natural killer cells (cor = 0.49, p < 0.001). FASN showed the most significant negative correlation with macrophages (cor = -0.48, p < 0.001) (**Figure 10D**). These results revealed abnormal immune cell infiltration patterns in bladder cancer, though derived from transcriptomic data without direct plasticizer exposure assessment, making their link to plasticizers indirect and hypothesis-generating.

## 4 Discussion

As additives widely present in daily life, plasticizers are commonly used in plastic products. Therefore, investigating the potential association between plasticizers and the risk of BCa can provide insights into the tumorigenesis of BCa and facilitate the development of more effective therapeutic strategies. Although direct causal evidence is absent, our in silico results support the hypothesis that chronic contact with commonly used plasticizers could hypothetically influence molecular events underlying BCa via pathways related to endocrine disruption, genotoxic stress, and immune dysregulation (27–29). In this study, seven potential candidate genes were identified by integrating multiple online databases and bioinformatics analyses. Subsequently, five key genes (CCNE1, KIT, BCL2, TGFBR2, and FASN) were identified through machine learning and expression validation. Furthermore, exploring the potential regulatory mechanisms of key genes involved in potential association between plasticizer exposure and BCa through methods such as GSEA and regulatory network construction offers new insights into the treatment of BCa.

These five core genes exert their biological functions primarily through two major regulatory modules: the regulation of cell cycle progression and the regulation of the PI3K-Akt signaling pathway. CCNE1 belongs to the cyclin family, and its encoded protein binds to CDK2 to regulate the G1-to-S phase transition of the cell cycle (39, 40). Accumulating evidence has confirmed that CCNE1 exhibits aberrant overexpression or gene amplification in various malignant tumors. Upregulated expression of this gene in bladder cancer accelerates aberrant cell cycle progression, promotes tumor cell proliferation, migration, and invasion, and is closely associated with poor patient prognosis and platinum-based chemotherapy resistance (41–43).

The remaining four core genes (KIT, BCL2, TGFBR2, and FASN) are all deeply involved in the signal transduction and functional regulation of the PI3K-Akt signaling pathway. Among them, KIT, as a receptor tyrosine kinase, binds to its ligand to activate downstream signaling and regulates cell proliferation, differentiation, and survival (44), and its dysfunction has been well documented in multiple tumors (45, 46). The anti-apoptotic protein BCL2 maintains cell survival by inhibiting mitochondrial apoptotic signaling, and its expression balance is also an important prognostic indicator for bladder cancer patients (47–49). TGFBR2 is a key upstream molecule of the TGF-β signaling pathway, participating in the regulation of cell growth and tumor microenvironment remodeling, and its aberrant expression can alter the malignant biological behaviors of bladder cancer cells (50–55). FASN, a key metabolic enzyme, is involved in the regulation of tumor lipid metabolism and can influence tumor progression through AKT-related signaling axes. Its high expression is significantly correlated with low survival rate and high recurrence risk in bladder cancer patients (56–58).

Overall, dysregulated expression of CCNE1 primarily leads to uncontrolled cell cycle progression, while aberrant expression of KIT, BCL2, TGFBR2, and FASN collectively induces aberrant activation of the PI3K-Akt signaling pathway, enhancing tumor cell survival and inhibiting apoptosis. The synergistic effect of cell cycle dysregulation and apoptosis blockade ultimately drives the initiation and malignant progression of bladder cancer. The results of our pathway enrichment analysis and GSEA also corroborate this mechanism. Meanwhile, numerous previous studies have confirmed that the PI3K-Akt signaling pathway occupies a central position in the development and progression of bladder cancer, further supporting the scientific validity and rationality of the plasticizer-core genes-signaling pathway regulatory axis constructed in this study (59, 60).

Research has shown that plasticizers can induce oxidative stress and disrupt endocrine functions, which are mechanisms that may contribute to carcinogenesis, including breast cancer and BCa. For instance, studies conducted on human placental cells have demonstrated that certain plasticizers can interfere with steroid synthesis and generate reactive oxygen species (ROS), which are known to cause cellular damage and contribute to cancer development (60). Additionally, the bioavailability of these chemicals in biological systems is a critical factor that influences their toxicity and potential to cause harm. Phthalate compounds may increase the risk of cancers such as prostate cancer and breast cancer by mimicking or interfering with hormonal functions (22). Furthermore, molecular docking results indicate that all five key genes can spontaneously bind with the three plasticizers. This finding suggests that plasticizers may participate in regulating immune processes by targeting these key genes, potentially through corresponding miRNAs or TFs.

Consistent with the urinary excretion theory described in the Introduction, plasticizers and their metabolites are primarily excreted via the urinary system, resulting in persistent exposure of the bladder urothelium to exogenous contaminants. This represents a critical reason why the bladder is more susceptible to toxic damage from endocrine-disrupting chemicals (61, 62). Through molecular docking and molecular dynamics simulations, this study found that three representative phthalates-DEP, DMP, and DOP-can stably bind to TGFBR2 and KIT receptor proteins, with all binding energies below-5.0 kcal/mol. These findings suggest that phthalates can alter the spatial conformation of membrane receptors and interfere with downstream signal transduction through physical binding. Among these receptors, TGFBR2 is a key regulator of urothelial proliferation, apoptosis, and epithelial-mesenchymal transition (63). Sustained disruption by phthalates can impair epithelial homeostasis and induce malignant transformation of cells.

Most importantly, this study is the first to reveal the potential role of KIT in phthalate-associated bladder cancer. While the biological function of KIT in bladder cancer remains poorly defined, our results demonstrate that KIT is downregulated in bladder cancer and forms stable complexes with phthalates. Integrating these findings with our GSEA results, we hypothesize that phthalates may target and inhibit KIT activity, disrupt cell cycle homeostasis, and promote malignant transformation of the bladder. In summary, leveraging the characteristic urinary accumulation and exposure, phthalates can regulate oncogenic signaling pathways by targeting and binding to TGFBR2 and KIT, thereby mediating the initiation and progression of bladder cancer. However, this receptor-mediated toxic mechanism requires further validation through subsequent *in vitro* exposure experiments and animal models.

Previous toxicological studies have demonstrated that phthalates possess well-documented immunotoxicity. They can disrupt the host’s adaptive immune responses, inhibit B lymphocyte activation and proliferation, and reduce the ability of immune cells to secrete inflammatory cytokines, thereby inducing an immunosuppressive state in the body (64, 65). The results of our ssGSEA for immune infiltration showed that the infiltration level of activated B cells was significantly downregulated in bladder cancer tissues, a trend highly consistent with the immunosuppressive characteristics mediated by phthalates. Based on these findings, we hypothesize that chronic phthalate exposure may suppress the B cell activation process, decrease the number of infiltrating activated B cells in the tumor microenvironment (TME), impair the host’s antitumor immune surveillance capacity, and create an immunosuppressive microenvironment conducive to the malignant progression of tumors. Notably, the daily urinary exposure concentrations of these compounds in the general population are relatively low, and humans are predominantly exposed to their metabolites. Therefore, the *in vitro* molecular docking binding effects observed in this study cannot fully represent the actual *in vivo* biological effects. This hypothesis requires further validation through subsequent experimental investigations.

Five genes (CCNE1, KIT, BCL2, TGFBR2, FASN) were identified. This study used bioinformatics and network pharmacology to generate hypotheses regarding the putative pathophysiology underlying the potential association between plasticizer exposure and BCa, offering insights into new therapeutic approaches that warrant further investigation. However, to further substantiate these findings, validation in larger sample cohorts and the application of more advanced research methods (such as animal model construction) will be necessary in the future.

Our ssGSEA analysis revealed that the infiltration of activated B cells was significantly lower in bladder cancer tissues than in normal control tissues. This observation preliminarily suggests a potential link between plasticizer exposure and immune dysregulation within the bladder tumor microenvironment. Toxicological studies have demonstrated that phthalate esters (e.g., DEHP and DEP) modulate B cell differentiation, suppress antibody production, and interfere with cytokine secretion via the PPAR signaling pathway and oxidative stress (66). Nevertheless, ssGSEA only infers immune enrichment based on transcriptomic profiles and cannot reflect the actual abundance and functional status of B cells. In addition, our samples lack documented records of plasticizer exposure. Accordingly, the proposed mechanistic axis of plasticizer exposure-B cell dysregulation-bladder cancer progression remains an indirect inference. Future investigations integrating quantitative exposure assessment, functional immunological assays (including flow cytometry), and *in vivo* animal models are required to validate these computational predictions.

When compared with existing prognostic markers for bladder cancer, our model yielded numerically comparable or slightly higher AUC values. Nevertheless, formal superiority testing was not performed, and any observed discrepancies should not be overinterpreted. The potential value of our research does not lie in claiming superior predictive performance, but in its distinctive biological rationale: it is specifically derived from genes associated with phthalate exposure and bladder cancer. This scoring model differs from traditional prognostic models that solely focus on the intrinsic biological characteristics of tumors. It exhibits remarkable environmental specificity, can effectively identify high-risk populations susceptible to chemical carcinogenic factors, and indirectly reflects the degree of tumor progression induced by exogenous chemical exposure. This model provides novel insights into the etiological classification and precision clinical intervention of environmental pollutant-associated bladder cancer. This signature may provide novel insights into environment-related molecular mechanisms underlying bladder cancer oncogenesis. Notably, the toxicity grades of DEP, DMP and DOP (Grade 4, 4 and 3, respectively) reflect their acute toxic potential rather than direct carcinogenicity. Nevertheless, the predicted capacity of these phthalates to target estrogen and androgen receptors warrants attention, as endocrine disruption has been recognized as one of the plausible mechanisms of bladder cancer development, functioning via hormone-mediated cellular proliferation and DNA damage (16). These in silico predictions merely serve as a basis for hypothesis generation, and require further experimental validation in bladder-specific model systems.

Several important limitations of the present study warrant explicit elaboration. First, This study specifically focuses on low-molecular-weight phthalates (DEP, DMP, and DOP) as representative environmental plasticizers. Although high-molecular-weight phthalates such as bis(2-ethylhexyl) phthalate (DEHP) are also extensively excreted via urine, they were excluded from the core network pharmacology analysis to streamline the research scope toward small-molecule compounds. Future investigations should incorporate high-molecular-weight plasticizers to comprehensively characterize the associations between phthalate congeners and bladder cancer. Second, Gene sets including PRGs and BCRTGs in this study were derived from public databases and published literature, wherein inconsistencies may exist regarding annotation quality and screening criteria. Identification of candidate genes is thus contingent upon the accuracy of upstream omics data. Although the T24 and SV-HUC-1 cell lines employed for *in vitro* validation are widely recognized models in bladder cancer research, they fail to fully recapitulate molecular heterogeneity and tumor microenvironment discrepancies among clinical patient samples. Accordingly, the present findings only provide preliminary supportive evidence. Likewise, the prognostic risk model constructed based on five core prognostic genes adopted the optimal cut-off value screened from the training cohort, leading to overoptimistic performance. The external validation AUC decreased from 0.71 to 0.65, indicative of potential overfitting. Hence, this model merely holds exploratory value and requires further validation across multiple independent cohorts before potential clinical translation. Moreover, we did not adjust for confounding factors such as smoking status, gender and treatment regimens due to data limitations, which may bring bias. Further prospective validation with environmental exposure data is still needed. Third, multiple testing correction was not implemented in the correlation analyses of immune infiltration, which may elevate the probability of spurious statistical significance. Fourth, the associations between plasticizers and bladder cancer in this study were exclusively inferred from computational predictions (network toxicology, molecular docking, and molecular dynamics simulations) combined with *in vitro* qRT-PCR validation in single cell lines. These results serve for hypothesis generation rather than causal verification. Without supporting evidence from epidemiological investigations or *in vivo* toxicological animal experiments, definitive conclusions regarding carcinogenic induction cannot be drawn. Fifth, despite the overall stable protein-ligand interactions revealed by the 100 ns MD simulation, long-timescale conformational transitions cannot be characterized, and rigorous quantitative estimation of binding affinity is not achievable. Sixth, we only verified basal gene expression in untreated cell lines and did not perform *in vitro* plasticizer exposure experiments, so the direct regulatory effect of plasticizers on target genes cannot be confirmed. The relevant results are merely preliminary hypotheses needing further experimental verification. Seventh, All datasets used in this study were retrieved from public repositories including TCGA and GEO. Owing to batch heterogeneity across sequencing platforms and specimen cohorts, the genomic inflation factor λ exceeded 1.05, indicating the presence of statistical inflation bias. Conventional correction approaches failed to eliminate such bias after two rounds of validation using genome-wide genes and differentially expressed genes (DEGs), which compromised the stability of differentially expressed gene screening and constitutes a major limitation of the present research. In follow-up work, in-house clinical specimens will be adopted with standardized sequencing protocols and optimized statistical correction strategies to reduce the inflation factor λ. Eighth, constrained by available data, confounding factors including smoking status, gender and therapeutic regimens were not adjusted for in the current analysis, which may introduce potential analytical bias. Further prospective validations incorporating individual environmental exposure data are therefore warranted in subsequent research. Therefore, current MD outcomes remain preliminary and hypothesis-generating. Future investigations are required to conduct stringent experimental validations, encompassing CRISPR screening, Pull-down assays, functional assays of cells upon plasticizer exposure, and *in vivo* animal model experiments.

## 5 Conclusion

This study integrates transcriptomics with network toxicology, providing a comprehensive framework for generating hypotheses about how plasticizers disrupt biomolecular networks and cellular functions, potentially influencing the development of BCa. To validate these hypotheses, the research further combines bioinformatics approaches with machine learning to identify five key genes (CCNE1, KIT, BCL2, TGFBR2, and FASN) that are putatively associated with both plasticizer exposure and BCa pathogenesis. These findings provide valuable insights that may have a profound impact on the treatment and prevention of BCa. However, it is important to note that the exploratory prognostic model presented herein requires additional validation prior to clinical translation.

## Data Availability

The datasets analysed in this study are available in the GEO database (http://www.ncbi.nlm.nih.gov/geo/) and the University of California, Santa Cruz (UCSC) Xena database (https://xena.ucsc.edu/), including GSE13507 and TCGA-BLCA. All processed analysis files generated during this study are available from the corresponding author upon reasonable request. No restrictions apply to data access.

## Supplementary material

Supplementary Figure 1 Kaplan–Meier survival curves of individual prognostic genes in the GSE13507 cohort. **(A)** CCNE1. **(B)** KIT. **(C)** BCL2. **(D)** TGFBR2. **(E)** FASN.

## References

[1] Jubber I Ong S Bukavina L Black PC Compérat E Kamat AM . Epidemiology of bladder cancer in 2023: a systematic review of risk factors. Eur Urol. (2023) 84:176–90. doi: 10.1016/j.eururo.2023.03.029 37198015

[2] Filho AM Briganti A Jemal A Bray F . Bladder cancer incidence and mortality: a global overview and recent trends. Eur Urol. (2026) 89:426–36. doi: 10.1016/j.eururo.2025.12.011 41421927

[3] Cumberbatch MGK Jubber I Black PC Esperto F Figueroa JD Kamat AM . Epidemiology of bladder cancer: a systematic review and contemporary update of risk factors in 2018. Eur Urol. (2018) 74:784–95. doi: 10.1016/j.eururo.2018.09.001 30268659

[4] Alfred Witjes J Max Bruins H Carrión A Cathomas R Compérat E Efstathiou JA . European association of urology guidelines on muscle-invasive and metastatic bladder cancer: summary of the 2023 guidelines. Eur Urol. (2024) 85:17–31. doi: 10.1016/j.eururo.2023.08.016 37858453

[5] Cin M Akyildiz IA Bektas S Gundogar O Cin S Komut N . Is immunohistochemical galectin-3 expression associated with the epithelial-mesenchymal transition in high- and low-grade invasive urothelial carcinomas of the bladder? Diagn (Basel). (2024) 14(20):2270. doi: 10.3390/diagnostics14202270 39451592 PMC11506668

[6] Li K Li S Tang S Zhang M Ma Z Wang Q . Kif22 promotes bladder cancer progression by activating the expression of cdca3. Int J Mol Med. (2021) 48(6):211. doi: 10.3892/ijmm.2021.5044 34633053 PMC8522959

[7] Babjuk M Burger M Capoun O Cohen D Comperat EM Dominguez EJ . European association of urology guidelines on non-muscle-invasive bladder cancer (ta, t1, and carcinoma in situ). Eur Urol. (2022) 81:75–94. doi: 10.1016/j.eururo.2021.08.010 34511303

[8] Robertson AG Kim J Al-Ahmadie H Bellmunt J Guo G Cherniack AD . Comprehensive molecular characterization of muscle-invasive bladder cancer. Cell. (2017) 171:540–56. doi: 10.1016/j.cell.2017.09.007 28988769 PMC5687509

[9] Choi W Ochoa A Mcconkey DJ Aine M Höglund M Kim WY . Genetic alterations in the molecular subtypes of bladder cancer: illustration in the cancer genome atlas dataset. Eur Urol. (2017) 72:354–65. doi: 10.1016/j.eururo.2017.03.010 28365159 PMC5764190

[10] Han S Li Y Chen D Si Z Xu T Du Y . Comprehensive genetic profile of chinese muscle-invasive bladder cancer cohort. Clin Genitourin Cancer. (2025) 23:102280. doi: 10.1016/j.clgc.2024.102280 39817975

[11] Net S Sempere R Delmont A Paluselli A Ouddane B . Occurrence, fate, behavior and ecotoxicological state of phthalates in different environmental matrices. Environ Sci Technol. (2015) 49:4019–35. doi: 10.1021/es505233b 25730609

[12] Zhang Y Guo J Xue J Bai C Guo Y . Phthalate metabolites: characterization, toxicities, global distribution, and exposure assessment. Environ pollut (Bark Essex 1987). (2021) 291:118106. doi: 10.1016/j.envpol.2021.118106 34520948

[13] Ren J Zhu Q Liao C Jiang G . Exposure levels and health risks of phthalate alternatives: a urinary metabolite study. Environ Health (Washington DC). (2025) 3:1175–86. doi: 10.1021/envhealth.5c00080 41127843 PMC12538321

[14] Liu S Guo W Qian Y Gu S Zheng J Xu K . Food-associated phthalate exposure and health risk: insights from urinary biomonitoring and dose-response modeling. Environ Sci Technol. (2025) 59:25613–22. doi: 10.1021/acs.est.5c09831 41269908

[15] Ple Nik H Rekar A Stevanovi S Virant-Klun I Imamovi Kumali S Sladi M . Nontargeted urinary profiling strategy for endocrine-disrupting chemicals in women with ovarian Malignancies. Environ Sci Technol. (2025) 59:8380–90. doi: 10.1021/acs.est.4c13290 40263667 PMC12060279

[16] Wang Y Ni H Dong S Shang Y Cui Z Zhu X . Environmental dose meohp promotes bladder cancer progress through hybrid emt mechanism: based on the adverse outcome pathway. Environ Sci Technol. (2026) 60:5979–90. doi: 10.1021/acs.est.5c08003 41650175

[17] Yin Z Jin X Mao C Kang L Gong C Feng S . Analysis of toxicity and mechanisms of dehp in prostate cancer with network toxicology and molecular docking strategy. Int J Surg. (2025) 111:1454–7. doi: 10.1097/JS9.0000000000001857 38905496 PMC11745634

[18] Paggi JM Pandit A Dror RO . The art and science of molecular docking. Annu Rev Biochem. (2024) 93:389–410. doi: 10.1146/annurev-biochem-030222-120000 38594926 PMC13198409

[19] Cheng M Li M Zhang Y Gu X Gao W Zhang S . Exploring the mechanism of ppcps on human metabolic diseases based on network toxicology and molecular docking. Environ Int. (2025) 196:109324. doi: 10.1016/j.envint.2025.109324 39952201

[20] Tan Z Chen X Zuo J Fu S Wang H Wang J . Comprehensive analysis of scrna-seq and bulk rna-seq reveals dynamic changes in the tumor immune microenvironment of bladder cancer and establishes a prognostic model. J Transl Med. (2023) 21:223. doi: 10.1186/s12967-023-04056-z 36973787 PMC10044739

[21] Gong M Song E Huang G Ni W Dong W Yuan R . Enhanced expression of cntd2/ccnp predicts poor prognosis in bladder cancer based on the gse13507. Front Genet. (2021) 12:579900. doi: 10.3389/fgene.2021.579900 33613629 PMC7886781

[22] He N Zhang J Liu M Yin L . Elucidating the mechanism of plasticizers inducing breast cancer through network toxicology and molecular docking analysis. Ecotox Environ Saf. (2024) 284:116866. doi: 10.1016/j.ecoenv.2024.116866 39178760

[23] Zheng Y Gao W Zhang Q Cheng X Liu Y Qi Z . Ferroptosis and autophagy-related genes in the pathogenesis of ischemic cardiomyopathy. Front Cardiovasc Med. (2022) 9:906753. doi: 10.3389/fcvm.2022.906753 35845045 PMC9279674

[24] Love MI Huber W Anders S . Moderated estimation of fold change and dispersion for rna-seq data with deseq2. Genome Biol. (2014) 15:550. doi: 10.1186/s13059-014-0550-8 25516281 PMC4302049

[25] Ito K Murphy D . Application of ggplot2 to pharmacometric graphics. CPT Pharmacometr Syst Pharmacol. (2013) 2:e79. doi: 10.1038/psp.2013.56 24132163 PMC3817376

[26] Gu Z Eils R Schlesner M . Complex heatmaps reveal patterns and correlations in multidimensional genomic data. Bioinformatics. (2016) 32:2847–9. doi: 10.1093/bioinformatics/btw313 27207943

[27] Wu T Hu E Xu S Chen M Guo P Dai Z . Clusterprofiler 4.0: a universal enrichment tool for interpreting omics data. Innovation (Camb). (2021) 2:100141. doi: 10.1016/j.xinn.2021.100141 34557778 PMC8454663

[28] Tay JK Narasimhan B Hastie T . Elastic net regularization paths for all generalized linear models. J Stat Softw. (2023) 106:1. doi: 10.18637/jss.v106.i01 37138589 PMC10153598

[29] Zhang Z Zhao Y Canes A Steinberg D Lyashevska O . Predictive analytics with gradient boosting in clinical medicine. Ann Transl Med. (2019) 7:152. doi: 10.21037/atm.2019.03.29 31157273 PMC6511546

[30] Fan Z Yang L Chen Y Wan W Zhou D Mai H . Prognostic significance of mrd and its correlation with arsenic concentration in pediatric acute promyelocytic leukemia: a retrospective study by scclg-apl group. Ther Adv Hematol. (2025) 16:1574229534. doi: 10.1177/20406207241311774 39781038 PMC11707783

[31] Zhang H Meltzer P Davis S . Rcircos: an r package for circos 2d track plots. BMC Bioinf. (2013) 14:244. doi: 10.1186/1471-2105-14-244 23937229 PMC3765848

[32] Orifjon S Jammatov J Sousa C Barros R Vasconcelos O Rodrigues P . Translation and adaptation of the adult developmental coordination disorder/dyspraxia checklist (adc) into asian Uzbekistan. Sports (Basel). (2023) 11(7):135. doi: 10.3390/sports11070135 37505622 PMC10383954

[33] Hakobyan S Boily JF Ramstedt M . Proton and gallium(iii) binding properties of a biologically active salicylidene acylhydrazide. J Inorg Biochem. (2014) 138:9–15. doi: 10.1016/j.jinorgbio.2014.04.012 24837332

[34] Trott O Olson AJ . Autodock vina: improving the speed and accuracy of docking with a new scoring function, efficient optimization, and multithreading. J Comput Chem. (2010) 31:455–61. doi: 10.1002/jcc.21334 19499576 PMC3041641

[35] Seeliger D de Groot BL . Ligand docking and binding site analysis with pymol and autodock/vina. J Comput-Aided Mol Des. (2010) 24:417–22. doi: 10.1007/s10822-010-9352-6 20401516 PMC2881210

[36] Kieninger S Keller BG . Gromacs stochastic dynamics and baoab are equivalent configurational sampling algorithms. J Chem Theory Comput. (2022) 18:5792–8. doi: 10.1021/acs.jctc.2c00585 36112147

[37] Charoentong P Finotello F Angelova M Mayer C Efremova M Rieder D . Pan-cancer immunogenomic analyses reveal genotype-immunophenotype relationships and predictors of response to checkpoint blockade. Cell Rep. (2017) 18:248–62. doi: 10.1016/j.celrep.2016.12.019 28052254

[38] Hanzelmann S Castelo R Guinney J . Gsva: gene set variation analysis for microarray and rna-seq data. BMC Bioinf. (2013) 14:7. doi: 10.1186/1471-2105-14-7 23323831 PMC3618321

[39] Zeng J Hills SA Ozono E Diffley J . Cyclin e-induced replicative stress drives p53-dependent whole-genome duplication. Cell. (2023) 186:528–42. doi: 10.1016/j.cell.2022.12.036 36681079 PMC7619399

[40] Zheng X Chen L Liu W Zhao S Yan Y Zhao J . Ccne1 is a predictive and immunotherapeutic indicator in various cancers including ucec: a pan-cancer analysis. Hereditas. (2023) 160:13. doi: 10.1186/s41065-023-00273-0 36964635 PMC10037856

[41] Kuhn E Bahadirli-Talbott A Shih I . Frequent ccne1 amplification in endometrial intraepithelial carcinoma and uterine serous carcinoma. Mod Pathol. (2014) 27:1014–9. doi: 10.1038/modpathol.2013.209 24309323

[42] Gorski JW Ueland FR Kolesar JM . Ccne1 amplification as a predictive biomarker of chemotherapy resistance in epithelial ovarian cancer. Diagn (Basel). (2020) 10(5):279. doi: 10.3390/diagnostics10050279 32380689 PMC7277958

[43] Matsushita R Seki N Chiyomaru T Inoguchi S Ishihara T Goto Y . Tumour-suppressive microrna-144-5p directly targets ccne1/2 as potential prognostic markers in bladder cancer. Br J Cancer. (2015) 113:282–9. doi: 10.1038/bjc.2015.195 26057453 PMC4506384

[44] Tsai M Valent P Galli SJ . Kit as a master regulator of the mast cell lineage. J Allergy Clin Immunol. (2022) 149:1845–54. doi: 10.1016/j.jaci.2022.04.012 35469840 PMC9177781

[45] Zhou S Abdihamid O Tan F Zhou H Liu H Li Z . Kit mutations and expression: current knowledge and new insights for overcoming im resistance in gist. Cell Commun Signal. (2024) 22:153. doi: 10.1186/s12964-023-01411-x 38414063 PMC10898159

[46] Cai B Liu Y Chong Y Mori SF Matsunaga A Zhang H . A truncated derivative of fgfr1 kinase cooperates with flt3 and kit to transform hematopoietic stem cells in syndromic and de novo aml. Mol Cancer. (2022) 21:156. doi: 10.1186/s12943-022-01628-3 35906694 PMC9336057

[47] Croce CM Vaux D Strasser A Opferman JT Czabotar PE Fesik SW . The bcl-2 protein family: from discovery to drug development. Cell Death Diff. (2025) 32(8):1369–1381. doi: 10.1038/s41418-025-01481-z 40204952 PMC12325696

[48] Thomalla D Beckmann L Grimm C Oliverio M Meder L Herling CD . Deregulation and epigenetic modification of bcl2-family genes cause resistance to venetoclax in hematologic Malignancies. Blood. (2022) 140:2113–26. doi: 10.1182/blood.2021014304 35704690 PMC10653032

[49] Golestani EB Sanati MH Houshmand M Ataei M Akbarian F Shakhssalim N . Expression and prognostic significance of bcl-2 and bax in the progression and clinical outcome of transitional bladder cell carcinoma. Cell J. (2014) 15:356–63. PMC3866540 24381861

[50] Fix SM Forget MA Sakellariou-Thompson D Wang Y Griffiths TM Lee M . Crispr-mediated tgfbr2 knockout renders human ovarian cancer tumor-infiltrating lymphocytes resistant to tgf-beta signaling. J Immunother Cancer. (2022) 10(7):e003750. doi: 10.1136/jitc-2021-003750 35882447 PMC9330322

[51] Fang Z Zhang N Yuan X Xing X Li X Qin X . Gabpa-activated tgfbr2 transcription inhibits aggressiveness but is epigenetically erased by oncometabolites in renal cell carcinoma. J Exp Clin Cancer Res. (2022) 41:173. doi: 10.1186/s13046-022-02382-6 35549739 PMC9097325

[52] Tschernia NP Gulley JL . Tumor in the crossfire: inhibiting tgf-beta to enhance cancer immunotherapy. BioDrugs. (2022) 36:153–80. doi: 10.1007/s40259-022-00521-1 35353346 PMC8986721

[53] Li T Wang H Xu J Li C Zhang Y Wang G . Tgfbr2 mutation predicts resistance to immune checkpoint inhibitors in patients with non-small cell lung cancer. Ther Adv Med Oncol. (2021) 13:17516787. doi: 10.1177/17588359211038477 34408796 PMC8366138

[54] Liu X Wu Y Zhou Z Huang M Deng W Wang Y . Corrigendum] celecoxib inhibits the epithelial-to-mesenchymal transition in bladder cancer via the mirna-145/tgfbr2/smad3 axis. Int J Mol Med. (2023) 52(2):64. doi: 10.3892/ijmm.2023.5267 31198976 PMC6605707

[55] Li Y Qiao L Zang Y Ni W Xu Z . Circular rna foxo3 suppresses bladder cancer progression and metastasis by regulating mir-9-5p/tgfbr2. Cancer Manag Res. (2020) 12:5049–56. doi: 10.2147/CMAR.S253412 32612392 PMC7323812

[56] Xu S Wu X Wang S Xu M Fang T Ma X . Trim56 protects against nonalcoholic fatty liver disease by promoting the degradation of fatty acid synthase. J Clin Invest. (2024) 134(5):e166149. doi: 10.1172/JCI166149 38206764 PMC10904058

[57] Su WT Chen JY Sun JB Huang QI Ke ZB Chen SH . Fatty acid metabolism-related molecular subtypes and a novel model for predicting prognosis in bladder cancer patients. J Biosci. (2024) 49:11. doi: 10.1007/s12038-023-00383-x 38186002

[58] Tao T Su Q Xu S Deng J Zhou S Zhuang Y . Down-regulation of pkm2 decreases fasn expression in bladder cancer cells through akt/mtor/srebp-1c axis. J Cell Physiol. (2019) 234:3088–104. doi: 10.1002/jcp.27129 30221356

[59] Li Y Cheng X Yan J Jiang S . Cthrc1 facilitates bladder cancer cell proliferation and invasion through regulating the pi3k/akt signaling pathway. Arch Med Sci. (2022) 18:183–94. doi: 10.5114/aoms.2019.85718 35154539 PMC8827022

[60] Li Y Guo G Song J Cai Z Yang J Chen Z . B7-h3 promotes the migration and invasion of human bladder cancer cells via the pi3k/akt/stat3 signaling pathway. J Cancer. (2017) 8:816–24. doi: 10.7150/jca.17759 28382144 PMC5381170

[61] Chou C Shu K Chen H Wang M Chang C Hsu B . Urine phthalate metabolites are associated with urothelial cancer in chronic kidney disease patients. Chemosphere. (2021) 273:127834. doi: 10.1016/j.chemosphere.2020.127834 33077191

[62] Meng M Yang Y Song L Peng J Li S Gao Z . Association between urinary phthalates and phthalate metabolites and cancer risk: a systematic review and meta-analysis. Heliyon. (2024) 10:e29684. doi: 10.1016/j.heliyon.2024.e29684 38665549 PMC11044039

[63] Tan M Zhang D Zhang E Xu D Liu Z Qiu J . Senp2 suppresses epithelial-mesenchymal transition of bladder cancer cells through desumoylation of tgf-βri. Mol Carcinog. (2017) 56:2332–41. doi: 10.1002/mc.22687 28574613

[64] Hansen JF Nielsen CH Brorson MM Frederiksen H Hartoft-Nielsen M Rasmussen ÅK . Influence of phthalates on *in vitro* innate and adaptive immune responses. PloS One. (2015) 10:e131168. doi: 10.1371/journal.pone.0131168 26110840 PMC4482536

[65] Martins K Hagedorn B Ali S Kennish J Applegate B Leu M . Tissue phthalate levels correlate with changes in immune gene expression in a population of juvenile wild salmon. Arch Environ Contam Toxicol. (2016) 71:35–47. doi: 10.1007/s00244-016-0283-7 27177745

[66] Zhuang Z You L Kong J Tian Y Li Y Zhi Y . Ppar subtypes determine distinct modes of action of phthalate esters (paes) and per- and polyfluoroalkyl substances (pfas) in disrupting human macrophage alternative activation. Toxicology. (2026) 521:154387. doi: 10.1016/j.tox.2025.154387 41456666

